# ReLink: Reliable Deep Learning for Microscopic Motion Tracking

**DOI:** 10.1002/advs.78541

**Published:** 2026-10-28

**Authors:** Yuan Jiang, Xiaochen Feng, Hongda Wei, Hao Sha, Hongpeng Wang, Min Zhang, Sanyang Han, Yongbing Zhang

**Affiliations:** ^1^ School of Computer Science and Technology Harbin Institute of Technology (Shenzhen) Shenzhen Guangdong China; ^2^ Institute of Biopharmaceutical and Health Engineering Tsinghua Shenzhen International Graduate School (SIGS) Tsinghua University Shenzhen Guangdong China

**Keywords:** reliable deep learning, single particle tracking, stochastic processes, trajectory linking

## Abstract

Single‐particle tracking (SPT) serves as a fundamental technique for deciphering complex microscopic dynamics in biological and physical systems by resolving trajectories of individual particles. However, linking localizations into trajectories remains challenging in realistic experiments, where crowding, blinking, and localization errors lead to linking ambiguity. Both statistical and deep‐learning approaches have been explored in these conditions, yet reliable link selection that generalizes across varying data distributions remains elusive. Here, we propose ReLink, a general deep‐learning framework for reliable trajectory linking in SPT. By integrating physical priors into geometric representations and leveraging confidence‐based adaptive filtering at inference, ReLink enables automated selection of trustworthy linkages, thereby preserving the fidelity of downstream trajectory‐based analyses. Across challenging simulated scenarios spanning broad experimental conditions and motion regimes, ReLink consistently outperforms representative statistical and deep learning‐based trajectory linking methods in both edge‐level and trajectory‐level evaluation metrics, leading to markedly improved downstream inference on public benchmarks. We further validate ReLink on diverse real‐world experiments, from free diffusion systems to crowded intracellular environments, demonstrating its practical significance for quantitative interpretation of microscopic dynamics and its promise for next‐generation trustworthy and automated SPT.

## Graphical abstract

ReLink enables reliable trajectory linking in single‐particle tracking by integrating physics‐informed motion statistics with confidence‐based adaptive filtering. Across diverse simulated and experimental conditions, ReLink robustly reconstructs trajectories and preserves the fidelity of downstream analyses, enabling accurate characterization of microscopic dynamics from physically verifiable free diffusion to complex intracellular anomalous diffusion.

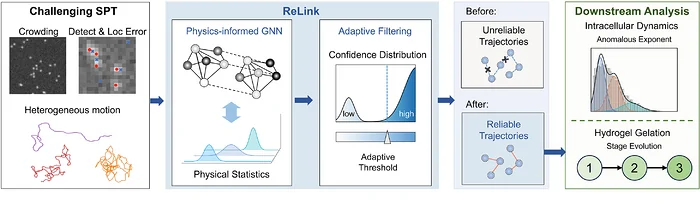

## 1 Introduction

The dynamics of molecules encode their interactions with the surrounding environment across micro‐ and nanoscales, thereby providing fundamental insights into a broad range of biological and physical processes [1, 2, 3, 4, 5]. Unlike ensemble‐averaged techniques like fluorescence correlation spectroscopy (FCS) [6], single‐particle tracking (SPT) leveraging optical microscopy has emerged as a pivotal technique for the direct observation of molecule or particle motions, uncovering fine‐scale dynamical mechanisms and environmental heterogeneity at the individual trajectory level [3, 7, 8, 9, 10, 11, 12]. This capability has inspired transformative applications, including characterization of molecular motor functions in the nervous system [13], elucidation of protein aggregation mechanisms in neurodegenerative diseases [14, 15], and mapping of viral infection pathways for precision drug discovery [16, 17]. To extract these insights, the standard tracking‐by‐detection SPT paradigm follows a sequential pipeline: particle localization, trajectory linking, and downstream trajectory analysis. In practical SPT experiments, however, beyond unavoidable particle localization errors, trajectory linking is complicated by stochastic heterogeneous particle motion such as time‐varying diffusion, anomalous transport, fusion or splitting events, and temporal dependencies, rendering it a structurally ill‐posed problem. Crucially, errors from trajectory linking can propagate into downstream trajectory analyses, distorting the characterization of particle dynamics and thereby potentially affecting the interpretation of the underlying biological processes [18]. Therefore, developing an accurate trajectory linking framework that generalizes across diverse scenarios remains critical in the SPT pipeline [19].

Numerous trajectory‐linking methods based on statistical assumptions about particle motion have been proposed to address this challenge [20, 21, 22, 23, 24, 25, 26, 27]. The simplest approaches rely on nearest‐neighbor association between detections in consecutive frames, which can be effective under low‐density and low‐speed conditions. As particle density increases, methods based on quadratic‐distance cost functions become more favorable. Specifically, a prominent class formulates trajectory linking as a Linear Assignment Problem (LAP), in which cost matrices are constructed from Euclidean distances and optimal assignments are obtained using the Hungarian algorithm [21, 22, 23]. Kalman filters have also been incorporated to iteratively predict particle positions under the assumption of constant velocity and Gaussian noise [25]. In more crowded scenarios, multiple‐hypothesis tracking methods reduce early‐decision bias by jointly considering multiple competing linkage hypotheses [26, 27]. However, these traditional statistics‐based approaches are highly sensitive to manually tuned hyperparameters and rely heavily on assumed motion models, thereby limiting their automated and consistently high‐accuracy performance across diverse scenarios.

With the rapid advancement of artificial intelligence, machine learning‐based approaches have emerged as compelling alternatives to traditional methods [28]. The Particle Tracking Challenge held in 2012 established a standardized benchmark for particle localization and linking, in which machine learning‐based methods demonstrated competitive performance across multiple experimental settings [24]. Building on superior hierarchical feature learning capabilities, deep learning‐based methods have further advanced trajectory‐linking accuracy [29, 30, 31, 32]. In particular, graph‐based formulations have gained attention in this context [31, 33, 34, 35], as they naturally capture the topology of particle linking. A representative example is MAGIK, which casts trajectory linking as a graph connectivity problem to predict candidate linkages and associated physical parameters [31]. Despite the high accuracy of deep learning‐based methods and their reduced reliance on manual parameter tuning, classical methods based on statistics and machine learning remain the prevailing choice among biologists. This preference partly stems from the black‐box nature of deep learning‐based methods and their uncertain behavior under distributional shifts between simulated training data and real‐world SPT data, including differences in particle dynamics, localization precision, and missed or false detections.

As deep learning generally encounters the potential threats from distributional shifts in practical applications, ensuring reliability is a critical aspect of deep learning‐based SPT technologies. Existing studies have attempted to address reliability from two complementary perspectives in deep learning‐based SPT. At the feature construction level, features derived from physically interpretable quantities (e.g., time‐averaged mean squared displacement and hidden Markov model states) as kind of prior information are explicitly incorporated to improve predictive interpretability and network transparency [36, 37]. At the inference level, well‐calibrated uncertainty estimates (e.g., via Bayesian deep learning and probabilistic modeling) in addition to predictions are introduced to elucidate the network's decision‐making process [38, 39]. These methods have been successfully applied to particle localization and downstream trajectory analysis, but their extension to trajectory linking remains challenging. As trajectory linking is inherently a combinatorial optimization problem, multiple competing linkage hypotheses across frames and incomplete observations can inevitably lead to ambiguity of linking [23]. To the best of our knowledge, there are few studies considering reliability in deep learning‐based trajectory linking, although incorrect trajectories can lead to severe errors in downstream analysis. As researchers generally prioritize trajectory correctness over quantity, it is essential to develop trajectory linking frameworks that preserve high linking accuracy as well as strong estimation of the output reliability.

Here, we present ReLink, a graph‐based deep learning framework for trajectory linking that integrates two key perspectives: physical statistics module and adaptive filtering module, to bridge the gap between accuracy and reliability. Specifically, the physical statistics module encodes jump‐distance priors to capture intrinsic particle dynamics, enriching geometric representations while preserving network transparency. At the inference stage, the adaptive filtering module augments linking predictions with confidence estimates and applies a data‐driven threshold to automatically select reliable linkages across both matched and shifted distributions. ReLink is evaluated against TrackMate [22], u‐Track [23], and Motion Transformer Tracker (MoTT) [29] and achieves stronger overall performance on simulated datasets spanning particle density, sequence length, motion dynamics, and imaging noise, as measured by the trajectory‐level Multiple Object Tracking Accuracy (MOTA) and edge‐level Edge F1 metrics. These improvements in trajectory reconstruction further translate into enhanced downstream analysis on the AnDi 1 and AnDi 2 benchmarks. Furthermore, three real‐world SPT experiments, including bead diffusion in glycerol‐water mixtures, diffusion‐state evolution during hydrogel gelation, and intracellular quantum‐dot tracking in L929 cells, demonstrate the robustness of ReLink, ensuring the fidelity of downstream analysis and supporting trustworthy SPT pipelines.

## 2 Results

### 2.1 Overview of ReLink

Trajectory linking in SPT experiments is challenged by diverse and compounding factors including high particle density, variable sequence length, localization noise, missed detections, and heterogeneous dynamics, which increase the ambiguity of candidate linkage hypotheses and the sensitivity of linking performance to experimental conditions (Figure 1a). As classical statistical methods rely on parameter tuning and existing deep learning‐based methods are limited by their black‐box nature, these methods can exhibit degraded and inconsistent performance on experimental data with unknown distribution, leading to unreliable trajectory linking. As shown in Figure 1b–d, our proposed framework couples a physics‐informed graph neural network (GNN) with a confidence‐based adaptive filtering module, improving the accuracy and reliability of trajectory linking (see Methods for details). Building upon the graph‐based representation that naturally captures linking topology [31], ReLink constructs a graph from localization data and distinguishes itself through two perspectives. To improve network transparency during feature construction, ReLink incorporates a physical statistics module that computes local and global jump‐distance histograms directly from each input localization sequence to encode particle spatiotemporal priors (Figure 1c). Jump distances are calculated from localization pairs at multiple temporal lags within predefined local or global spatial ranges, which can be adjusted for different experimental conditions. Local histograms characterize displacement patterns within the neighborhoods, whereas global histograms summarize the overall motion statistics across the full field of view (FOV). These histograms are computed without additional parameter optimization and incorporated into the graph representation through feature‐wise linear modulation (FiLM) [40] and multi‐head attention [41], allowing ReLink to selectively integrate motion information relevant to each candidate edge across temporal and spatial scales. Furthermore, to enhance reliability at the inference level, our framework employs an adaptive filtering module with Monte Carlo dropout [42] to estimate link‐level confidence along with linking probability (Figure 1d). The confidence scores of all predicted links in an input sequence form a confidence distribution, which is smoothed using kernel density estimation (KDE) [43] to characterize lower‐ and higher‐confidence regions. ReLink therefore automatically identifies the local minimum as a data‐driven adaptive threshold to filter out ambiguous linkages without manual tuning, thereby improving the robustness of trajectory reconstruction and downstream analysis. To evaluate ReLink under a consistent training protocol, the analyses presented below use a single model trained on a simulated high‐density dataset comprising 50‐frame multi‐state fractional Brownian motion (FBM) trajectories with multiple diffusion coefficients. No retraining or fine‐tuning is performed unless explicitly stated. For fair comparison, the same dataset‐specific gap‐closing setting is applied to ReLink and all applicable baseline methods (see Methods for details).

**FIGURE 1:**
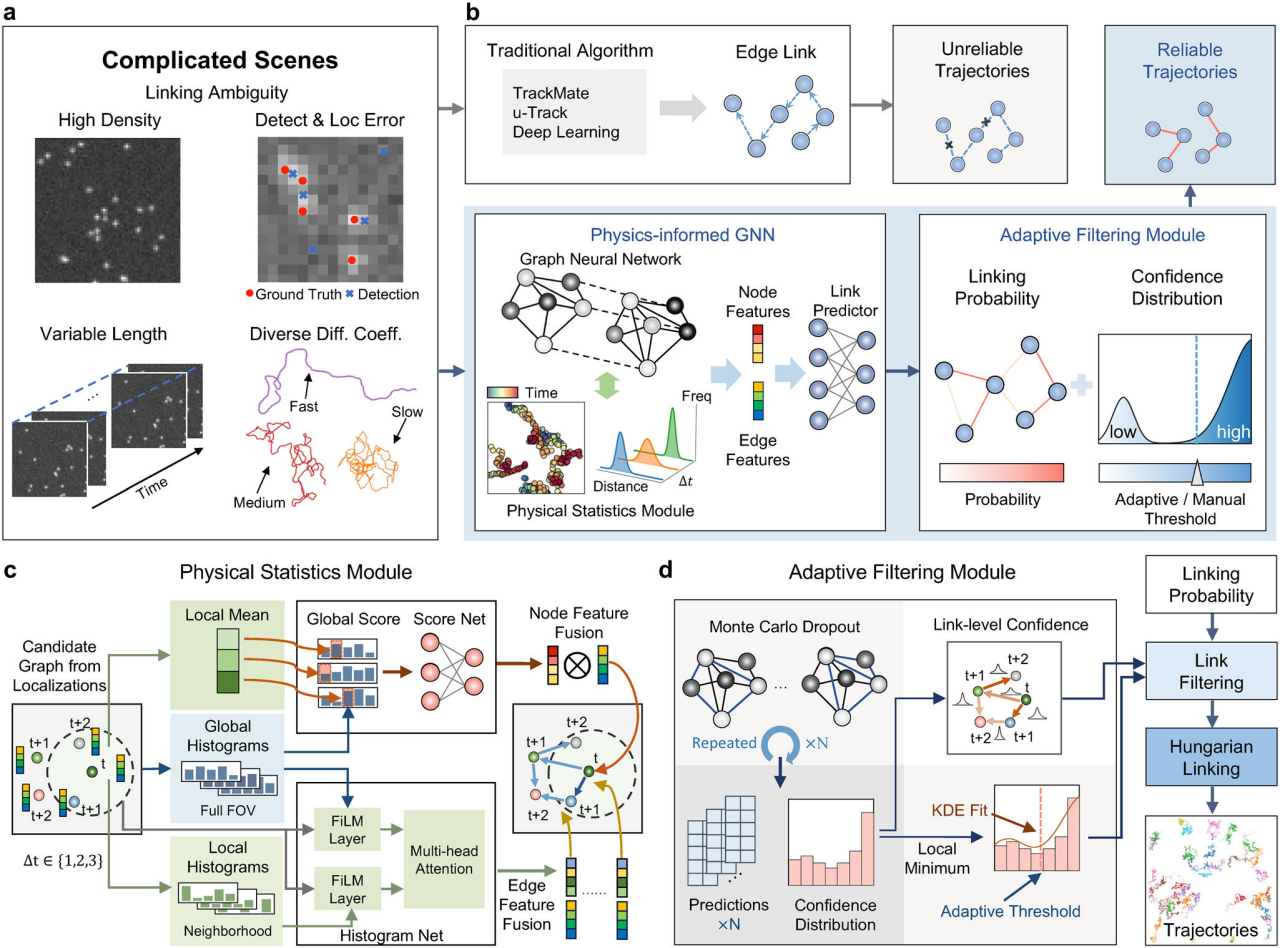
Overview of ReLink for reliable trajectory linking in single‐particle tracking. (a) Key challenges in real SPT trajectory linking, including high particle density, variable sequence length, missed detections, localization noise, and heterogeneous dynamics that induce ambiguous linking hypotheses. (b) Conceptual comparison of trajectory‐linking methods. Classical statistical methods (e.g., TrackMate and u‐Track) rely on hand‐tuned hyperparameters and assumed motion models, whereas existing deep learning approaches often operate as black boxes and can be unreliable under differences between training and experimental data. ReLink addresses this gap by combining a physics‐informed graph neural network (GNN) for accurate linkage prediction with a confidence‐based adaptive filtering module for decision‐level reliability. In ReLink, detections are represented as a spatiotemporal graph in which nodes correspond to particle localizations and candidate edges connect nearby detections based on spatial proximity and temporal separation. The GNN predicts a linking probability for each candidate edge. (c) Schematic of the physical statistics module. Local and global jump‐distance statistics are computed directly from all localization pairs satisfying the corresponding spatial constraints at temporal lags of ∆t = 1,2,3, without requiring prior knowledge of particle identities. Local statistics characterize motion within local neighborhoods, whereas global histograms summarize motion across the full field of view. The local and global histograms are integrated into the edge features through feature‐wise linear modulation (FiLM) and multi‐head attention. In parallel, the correspondence between local mean jump distances and the global distributions is encoded as scores, which are embedded by a Score Net and incorporated into the node features. Together, these physical statistics provide relevant multi‐scale motion information for linking prediction. (d) Illustration of the confidence‐based adaptive filtering module during inference. Monte Carlo dropout generates repeated stochastic linking predictions, from which link‐level confidence scores are estimated. The confidence scores form a confidence distribution that is smoothed using kernel density estimation (KDE), and the local minimum between its low‐ and high‐confidence modes defines an adaptive confidence threshold. Candidate links below this threshold are removed by confidence‐based link filtering, after which the linking probabilities of the retained candidates are used via Hungarian algorithm to reconstruct the final trajectories.

### 2.2 ReLink Achieves High‐Accuracy Trajectory Linking Across Diverse Simulations

To assess whether integrating a graph neural network with the physical statistics module enables robust and accurate linking at the feature‐construction stage, our method was benchmarked across a wide range of simulated particle‐diffusion scenarios, with the FOV set to 64 × 64 pixels by default unless otherwise specified. We first consider ideal conditions with perfect localization, where the influence of detection and localization errors is eliminated. This setting allows the intrinsic linking capability of the proposed method to be systematically evaluated across broad experimental variables. A representative example is shown in Figure 2a, where the field of view contains particles exhibiting heterogeneous diffusivities. Long trajectories of fast‐moving particles are accurately reconstructed by ReLink and more closely match the ground truth (GT), particularly in the boxed zoom region, where MoTT, TrackMate, and u‐Track exhibit clear linking errors. Across systematic variations in particle density and video sequence length (Figure 2b; Figures S1 and S2), ReLink maintains the highest linking performance. As density increases from 5 to 20 particles per frame, MOTA/Edge F1 decrease only from 0.995/0.995 to 0.977/0.979, while remaining significantly higher than those of all three baselines (Table S1). The linking performance of our method also maintains MOTA ≈ 0.99 across sequence length (20 to 200 frames) and remains stable across field‐of‐view scales (Figure 2c). Notably, ReLink achieves near‐perfect MOTA and Edge F1 on motion models absent from the training data, including single‐state motion, confinement with motion restricted to bounded regions, dimerization in which two particles transiently associate and move together, and temporary immobilization, demonstrating strong generalization to previously unseen motion dynamics (Figure 2c).

**FIGURE 2:**
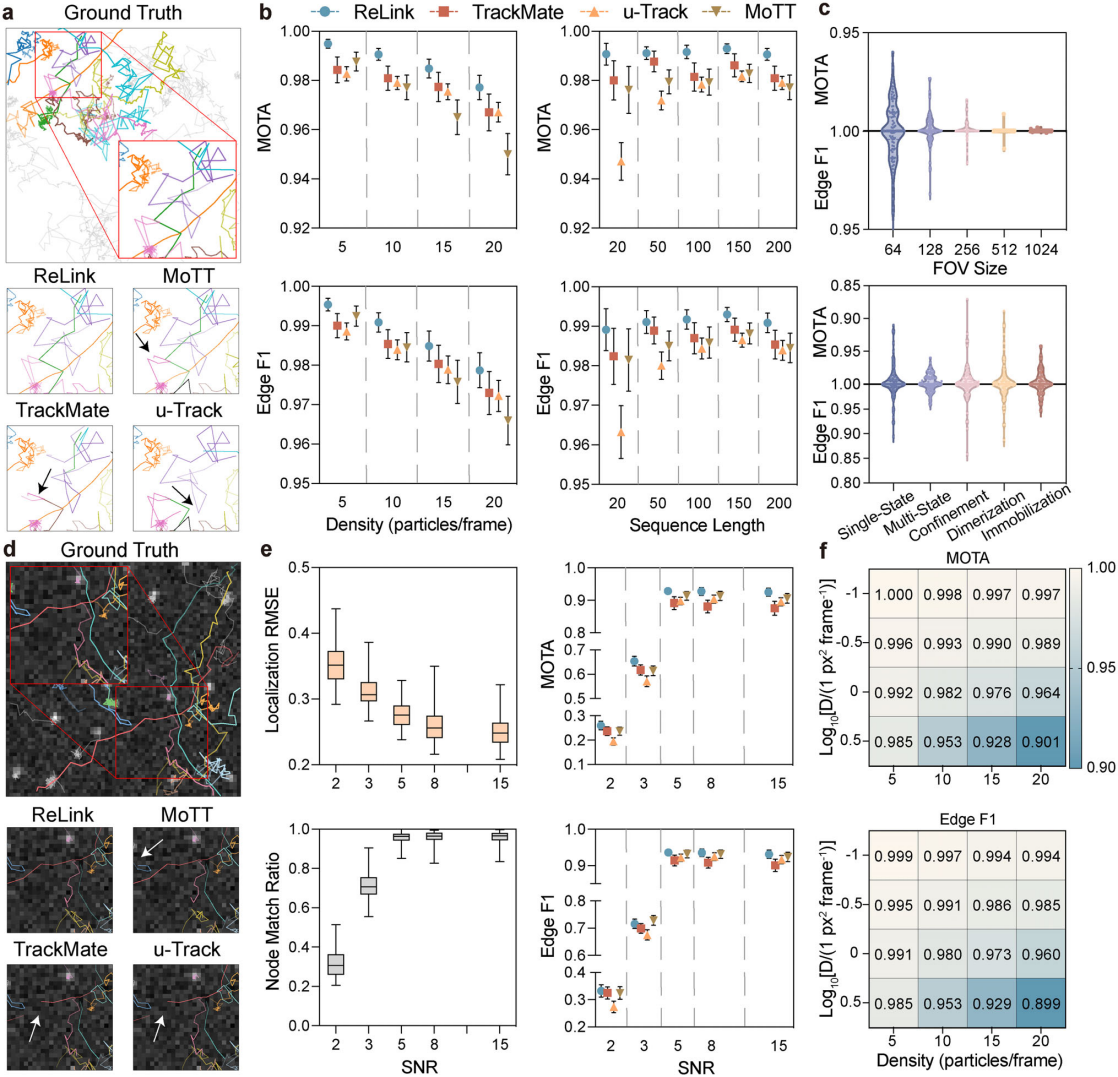
Comprehensive evaluation of trajectory linking performance on simulated data. (a) Representative trajectories (20 particles/frame; 200 frames) under ideal localization inputs, comparing ground truth (GT) with ReLink, MoTT, TrackMate and u‐Track. Black arrows highlight representative linking errors. (b) MOTA and Edge F1 under varying particle densities and sequence lengths. Particle number is varied from 5 to 20 particles/frame within a fixed 64 × 64‐pixel FOV, corresponding to particle densities of 1.22 × 10^−3^ to 4.88 × 10^−3^ particles/px^2^. Density experiments are performed with 200‐frame sequences, whereas sequence‐length experiments use 10 particles/frame. Data are presented as means with 95% confidence intervals (*n* = 55 independently simulated sequences per condition). (c) Evaluation of ReLink performance (MOTA and Edge F1) across different field‐of‐view sizes and particle motion models, including single‐state, multi‐state, confinement, dimerization, and immobilization. For the FOV‐size experiments, the particle number is fixed at 10 per frame across all FOV sizes. Violin plots show the distributions across 55 independently simulated sequences per condition. (d) Representative video‐based simulation at SNR = 5, showing the ground‐truth trajectories and corresponding reconstructions by ReLink, MoTT, TrackMate, and u‐Track. White arrows indicate representative linking errors under noisy imaging conditions. (e) Localization and trajectory‐linking performance across SNR = 2, 3, 5, 8, and 15, evaluated by localization RMSE (pixel), node match ratio, MOTA, and Edge F1. Localization RMSE and node match ratio characterize the localization inputs shared by all linking methods. Box plots show the median and interquartile range, with whiskers indicating the minimum and maximum values; MOTA and Edge F1 are presented as mean with 95% confidence intervals (*n* = 55 independently simulated sequences per condition). (f) Combined effects of particle density and diffusion coefficient on ReLink performance. Heatmaps show the mean MOTA and Edge F1 across density and diffusion coefficient D, expressed as log_10_[D/(1 px^2^ frame^−1^)]. Each condition contains *n* = 55 independently simulated sequences. Statistical comparisons of ReLink with TrackMate, u‐Track and MoTT in (b) and (e) are reported as Dunnett‐adjusted multiple comparisons in Tables S1 and S2, respectively. All comparisons across particle densities in (b) are statistically significant (adjusted *P* < 0.05). In (e), all comparisons are significant except for Edge F1 between ReLink and TrackMate at SNR = 2 (adjusted P = 0.0664). Unless otherwise specified, each sequence in the simulations contains 10 particles/frame, 50 frames, and diffusion coefficients ranging from 10^−1^ to 10^1^ px^2^/frame.

To move beyond idealized localization, we next evaluate ReLink under controlled localization and detection imperfections commonly encountered in SPT experiments. Specifically, localization errors, false detections, and missed detections are introduced independently while the remaining conditions are held fixed, allowing their individual effects on trajectory linking to be evaluated separately. A clear advantage is maintained under increasing false detections and mild‐to‐moderate localization uncertainty, whereas increasing missed detections (from 5% to 60%) leads to a more pronounced deterioration in trajectory linking performance for all methods (Figure S3). We then evaluate linking performance on simulated images with controlled noise levels, with signal‐to‐noise ratios (SNRs) ranging from 2 to 15, where the previously considered errors become coupled as image quality deteriorates. Figure 2d visualizes linking results at SNR of 5, where ReLink accurately reconstructs long trajectories even under severe noise (white arrow). Additional examples across SNR levels are shown in Figure S4. Quantitatively, ReLink consistently achieves higher MOTA than all baselines across the entire SNR range and higher Edge F1 in most comparisons (Figure 2e; Table S2). At SNR of 3, where only ≈ 70% of detections remain, MOTA/Edge F1 reach 0.654/0.716. Even at the extremely challenging SNR of 2 (≈ 30% of detections retained), ReLink still achieves the highest linking quality despite the substantial degradation experienced by all methods. Finally, we examine the combined effects of particle density and diffusion coefficient at SNR of 5, two factors that jointly increase linking ambiguity by increasing both the number of competing candidates and the displacement between successive detections (Figure 2f). ReLink maintains high MOTA and Edge F1 over most of the evaluated parameter space, with performance decreasing primarily under the most challenging combination of high density and rapid diffusion. These simulation experiments demonstrate the strong performance of ReLink across variations in particle density, video length, field‐of‐view scale, motion‐type diversity, and imaging noise. Enabled by the physical statistics module and its physically grounded feature construction, ReLink achieves robust and accurate candidate link prediction under challenging conditions, establishing the first layer of reliability for the subsequent adaptive filtering strategy.

### 2.3 ReLink Reveals Reliable Trajectories Through Confidence‐Based Adaptive Filtering

Building on the robust candidate linking established by the physical statistics module, we next address the residual ambiguity that persists at the individual link level. While overall linking accuracy is relatively high, a non‐negligible fraction of candidate links remains uncertain, necessitating explicit mechanisms for confidence‐aware link selection. Existing trajectory‐linking methods typically provide only binary linking decisions and offer limited estimates of predictive certainty. To address this limitation at the inference stage, ReLink augments candidate edge probabilities with an adaptive filtering module to support reliable link selections. Specifically, using Monte Carlo dropout, link‐level confidence is estimated by sampling multiple stochastic forward passes. The confidence scores of all predicted links are aggregated to form a confidence distribution. This resulting distribution is then fitted using kernel density estimation (KDE) and typically reveals a low‐confidence mode associated with ambiguous links and a high‐confidence mode associated with more reliable links. The local minimum between the two modes is used as an adaptive confidence threshold without manual tuning. Candidate edges with confidence values below this threshold are pruned, thereby improving the reliability of the reconstructed trajectories. ReLink's confidence mechanism is illustrated using a representative example (Figure 3a). Figure 3b shows a zoomed‐in view of the red‐boxed region in Figure 3a, comparing linking results across various fixed confidence thresholds and the adaptive threshold (Figure S5). As the threshold increases from 0.4, low‐confidence incorrect edges are gradually removed, and correct trajectory segments emerge. Conversely, overly strict thresholds (e.g., 0.9) may discard many true‐positive edges, leading to fragmented trajectories. Notably, the adaptive threshold consistently recovers high‐quality trajectories across settings. In the absence of the physical statistics module, more incorrect long‐range links and fragmented trajectories emerge, indicating that the physical statistics module supports confidence‐based reliable link selection. In this representative example, correct links are predominantly concentrated near a confidence of 1, whereas incorrect links are concentrated around 0.3–0.5, yielding an adaptive threshold of 0.60 (Figure 3c). On a larger test set, Figure 3d illustrates that increasing the confidence threshold progressively reduces the number of correctly matched edges, whereas MOTA initially increases and then declines as excessive filtering removes valid links. Relative to no filtering (dashed lines), the adaptive threshold and fixed thresholds from 0.4 to 0.8 significantly improve MOTA both with and without the physical statistics module (Table S3). Incorporating the physical statistics module further increases MOTA across all filtering settings (Table S4), with the adaptive threshold achieving performance within the top‐performing range of manually selected thresholds without manual tuning. Similar trends are observed in additional experiments with dimerization and confinement motion models (Figure S6), confirming the consistency of our method across different dynamical regimes.

**FIGURE 3:**
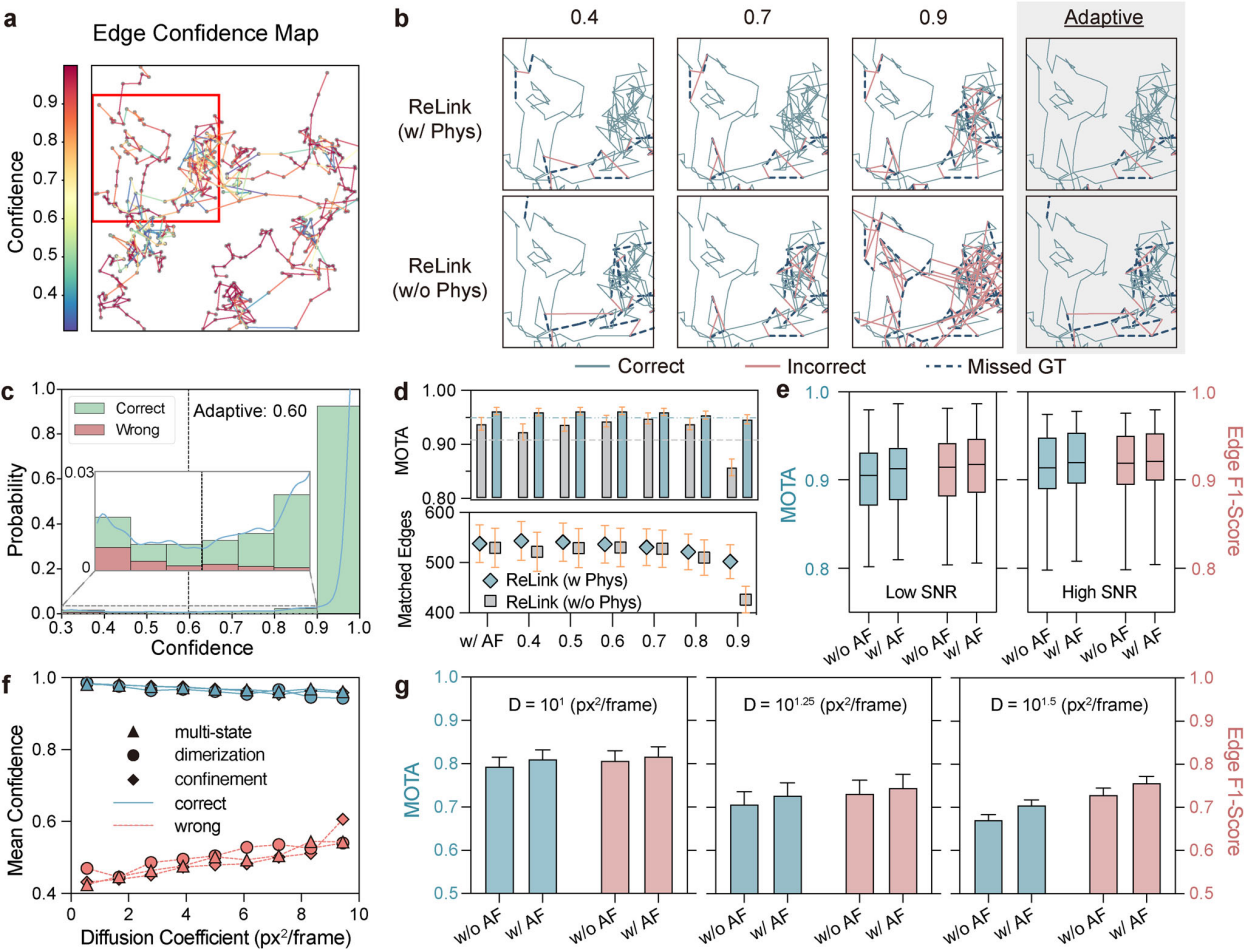
Confidence estimates and adaptive filtering for trajectory linking. (a) Visualization of confidence scores estimated by ReLink for candidate edges in a representative example. Edge colors encode confidence values, with red indicating higher confidence and blue indicating lower confidence. (b) Linking results in the red‐boxed region in (a) obtained using fixed confidence thresholds of 0.4, 0.7, and 0.9 or the adaptive threshold, with and without the physical statistics module (Phys). Correctly predicted links are shown in blue‐green, incorrect links in red, and missed ground‐truth links as dark‐blue dashed lines. (c) Confidence histograms of correct and incorrect predicted links in (a), overlaid with a kernel density estimate curve of the overall confidence distribution. The inset enlarges the low‐probability region. The vertical dashed line marks the local minimum of the density estimate, which gives an adaptive threshold of approximately 0.60. In this example, the adaptive filtering module removes 70.2% of the incorrect links while retaining 97.9% of the correct links, resulting in an accuracy of 99.5% among the retained links. (d) Quantitative evaluation of trajectory linking for the dataset containing the example in (a). MOTA and the number of correctly matched edges are shown across fixed confidence thresholds and the adaptive threshold, with and without the physical statistics module (Phys). Here, “w/ AF” denotes ReLink with the adaptive filtering module using its default adaptive threshold, while numerical labels indicate fixed confidence thresholds. Dashed lines represent the corresponding results without confidence‐based filtering (w/o AF). Each simulated sequence contains 15 particles/frame within 64 × 64‐pixel FOV (3.66 × 10^−3^ particles/px^2^), spans 50 frames, and includes diffusion coefficients ranging from 10^0^ to 10^1^ px^2^/frame. Data are presented as mean with 95% confidence intervals (*n* = 55 independently simulated sequences per setting). Dunnett‐adjusted comparisons of each confidence‐based filtering condition with unfiltered linking and Bonferroni‐adjusted comparisons between ReLink with and without the physical statistics module are provided in Tables S3 and S4, respectively. Comparisons with unfiltered linking are significant, except for the fixed threshold of 0.9 with Phys (adjusted P = 0.7055). All comparisons between ReLink with and without the physical statistics module are statistically significant (adjusted *P* < 0.05). (e) Trajectory‐linking metrics for challenging video‐based simulations from Figure 2 under low (SNR = 5) and high (SNR = 15) signal‐to‐noise ratios, with (w/ AF) and without (w/o AF) the adaptive filtering module. Box plots show the median, interquartile range, and minimum‐to‐maximum range (*n* = 30 for SNR = 5 and *n* = 24 for SNR = 15). Bonferroni‐adjusted multiple comparisons are provided in Table S5. (f) Mean confidence of correctly and incorrectly predicted links across diffusion coefficients for multi‐state, dimerization, and confinement motion models. The particle density, FOV size, sequence length, and diffusion‐coefficient range follow the same simulation settings as in (d), while the motion model is varied. (g) Trajectory‐linking performance metrics (MOTA and Edge F1) under extremely rapid particle motion at diffusion coefficient D = 10^1^, 10^1.25^, and 10^1.5^ px^2^/frame, comparing results obtained with (w/ AF) and without (w/o AF) the adaptive filtering module. Data are presented as mean with 95% confidence intervals (*n* = 55 independently simulated sequences per condition). Bonferroni‐adjusted multiple comparisons are provided in Table S6. All comparisons between w/ AF and w/o AF in (e) and (g) are statistically significant (Bonferroni‐adjusted *P* < 0.05).

We further evaluate the effectiveness of the adaptive filtering module under more challenging conditions involving diverse dynamics and noise. As shown in Figure 3f, the mean confidence values are compared across multi‐state, dimerization, and confinement motion models. In all cases, correctly predicted edges maintain high mean confidence values of above 0.9, whereas incorrectly predicted edges show substantially lower values of approximately 0.4–0.6. This separation is consistent with the bimodal confidence structure observed above and indicates that the confidence estimates remain discriminative across heterogeneous dynamics. In challenging video‐based simulations, significant improvements in both MOTA and Edge F1 are also observed after adaptive filtering under both low‐ and high‐SNR conditions (Figure 3e; Table S5). Finally, we extend the analysis to extremely fast diffusion regimes, where trajectory linking becomes substantially more ambiguous. Despite the increased linking difficulty, the adaptive filtering module continues to yield noticeable improvements (Figure 3g; Table S6). Overall, these results indicate that the physical statistics module enhances the separability of confidence estimates, while adaptive confidence filtering automatically retains high‐quality edges and improves final reconstruction quality. Together, these two components form a solid foundation for ReLink's superior performance in complex trajectory‐linking scenarios.

### 2.4 ReLink Demonstrates Strong Performance on Public Benchmarks

To further examine the impact of improved reliable trajectory linking on downstream trajectory analysis, ReLink was benchmarked on the AnDi 1 and AnDi 2 Challenge datasets [19, 44], following the official evaluation protocols and metrics (see the ‘Simulations’ section in the Methods). We construct a test set from the AnDi 1 Challenge [44] to assess the impact of improved linking on diffusion model classification and anomalous exponent (α) regression. Trajectories reconstructed with different linking methods are fed into the same downstream analysis networks [38] to infer diffusion type and anomalous exponent. As shown in Figure 4a, ReLink achieves better classification F1 and α‐regression mean absolute error (MAE), outperforming both traditional baselines and approaching the performance obtained with GT trajectories. Remarkably, although ReLink was trained only on FBM multi‐state data, it generalizes well across all five diffusion models in AnDi 1 (Figure S7). These findings indicate that ReLink's high‐quality linking serves as a critical prerequisite for more precise anomalous‐diffusion identification across heterogeneous diffusion models. To evaluate performance at both trajectory and ensemble levels, we replace the TrackMate linking step in U‐LFormer [45], a top‐performing method on the AnDi 2 Benchmark [19], with ReLink while keeping all downstream analysis components unchanged. As shown in Figure 4b, all major metrics improve substantially, reducing MSLE (D) by ≈ 50% while increasing JSC (CP) by ≈ 37% (Table S7). Figure 4c shows the comparison of performance across different confidence thresholds, consistent with our earlier observations: the adaptive threshold provides the most stable and robust improvement across both single‐trajectory and ensemble evaluations (Table S7). In the original AnDi 2 Challenge, the reference method used TrackMate for localization and linking together with U‐LFormer for trajectory analysis, ranking 3rd for single‐trajectory tasks and 1st for ensemble tasks in the video track. Our results suggest that only incorporating ReLink in place of the original linking step could substantially strengthen overall trajectory analysis performance.

**FIGURE 4:**
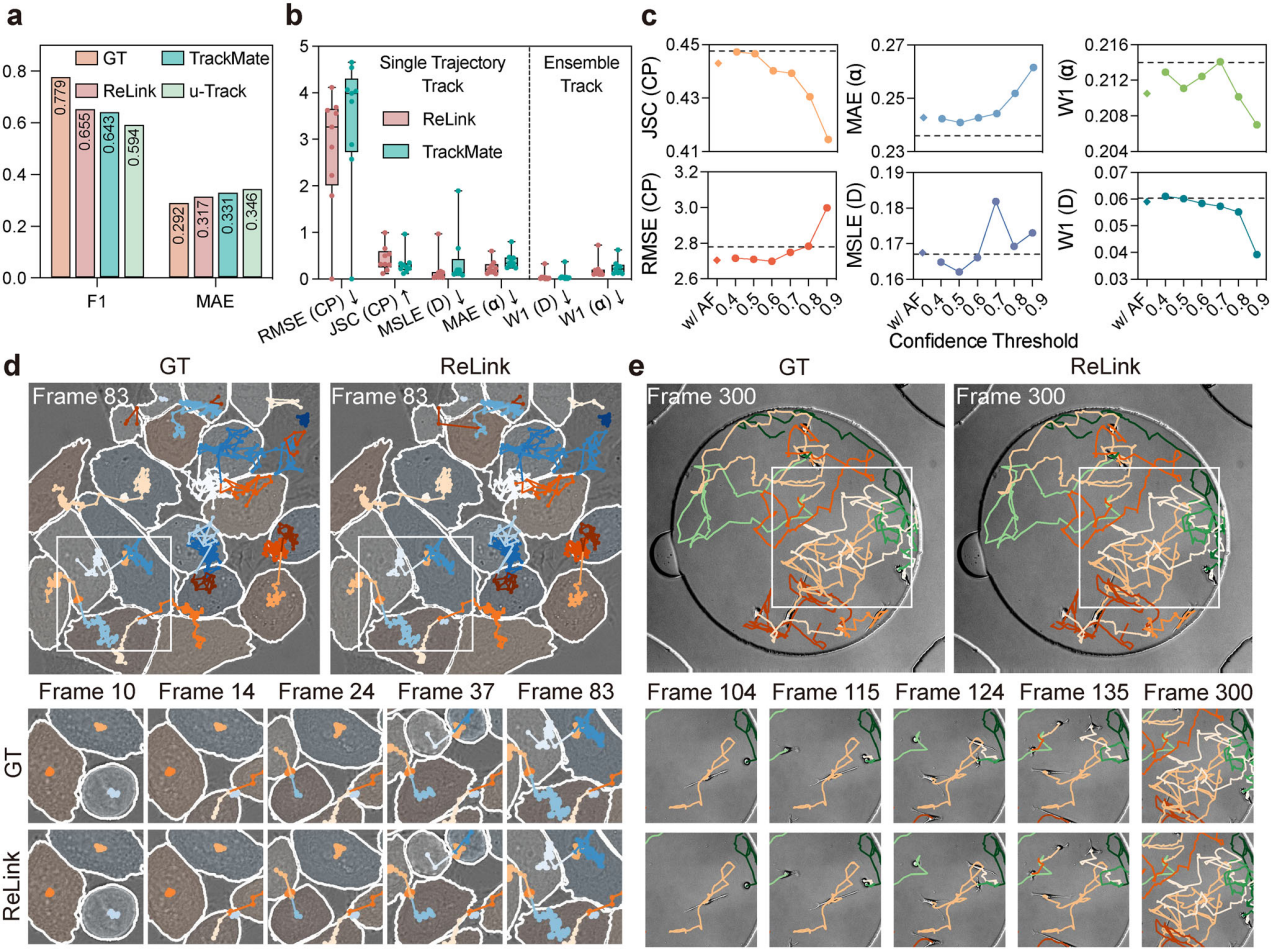
Enhanced public benchmark metrics through improved trajectory linking with ReLink. (a) Performance comparison on the test set constructed from AnDi 1 Benchmark. Diffusion‐model classification (F1 score) and anomalous‐exponent regression (MAE) are evaluated using trajectories reconstructed by ReLink, TrackMate, and u‐Track, as well as ground‐truth trajectories. (b) Evaluation on the AnDi 2 Benchmark Video Track. For the single‐trajectory task, we report the root mean square error of changepoint detection (RMSE (CP)), the Jaccard similarity coefficient for changepoint detection (JSC (CP)), the mean absolute error of the anomalous exponent (MAE (α)), and the mean squared logarithmic error of the diffusion coefficient (MSLE (D)). For the ensemble task, we report the Wasserstein‐1 distance of the anomalous exponent and diffusion coefficient distributions (W1 (α) and W1 (D)). Metrics are computed on trajectories reconstructed by TrackMate and ReLink with the adaptive filtering module. Box plots show the median and interquartile range; whiskers indicate the minimum and maximum, and points represent the nine individual experiments. (c) Influence of confidence‐based filtering on AnDi 2 Benchmark performance. Single‐trajectory metrics (left two columns) include JSC (CP), RMSE (CP), MAE (α), and MSLE (D). Ensemble metrics (right one column) include W1 (α) and W1 (D). Results are reported for no confidence filtering (dashed lines), the adaptive threshold (w/ AF), and fixed confidence thresholds (0.4–0.9). (d, e) Trajectory linking results on the Cell Tracking Challenge datasets DIC‐C2DH‐HeLa (d) and BF‐C2DL‐MuSC (e). Representative regions containing cell‐division events are indicated by white boxes, with enlarged views showing reconstructed trajectories before and after division.

Beyond single‐particle motion, cell trajectories represent an important class of biological dynamics with distinct characteristics and scientific relevance. We investigate whether ReLink is limited to FBM‐based particle motion or can generalize to biological motion patterns. Specifically, we evaluate ReLink on two datasets from the Cell Tracking Challenge [46]: DIC‐C2DH‐HeLa (Figure 4d) and BF‐C2DL‐MuSC (Figure 4e). Using detection coordinates provided by DeepTrack [47] (see Note S3 for dataset preparation details), the performance of our framework in linking and cell‐division detection can be assessed across cell types. The ReLink model, trained exclusively on simulated multi‐state FBM trajectories, is directly applied to the HeLa data without additional training or fine‐tuning. Although the simulated training data contain no explicit examples of cell division, the model correctly identifies division events and reconstructs the corresponding pre‐ and post‐division trajectories (Figure 4d). We next evaluate cross‐cell‐type transfer using limited annotated data. A separate ReLink model is trained on the labeled trajectory data from a single HeLa FOV and then tested on the MuSC data without MuSC‐specific fine‐tuning. Despite the different cell type and motion characteristics, this model accurately reconstructs the representative MuSC trajectories shown in Figure 4e. Quantitatively, ReLink achieves MOTA/Edge F1 values of 0.948/0.962 for HeLa and 0.982/0.977 for MuSC. These results demonstrate both zero‐shot transfer from simulated particle motion to cell tracking and cross‐cell‐type generalization from limited HeLa annotations.

### 2.5 ReLink Enables Reliable Diffusion Analysis in Real‐World SPT Systems

Although ReLink achieves strong performance on simulations and public benchmarks, its effectiveness under realistic experimental conditions remains to be established. In practice, finite temporal resolution and motion blur, together with background autofluorescence, out‐of‐focus fluorescence, and signal overlap in crowded fields, can compromise particle detection and localization, thereby increasing ambiguity in subsequent trajectory linking [48, 49, 50, 51]. To assess the practical impact of reliable trajectory reconstruction across various conditions, we evaluate ReLink in three real‐world experimental systems with increasing environmental heterogeneity.

#### 2.5.1 Brownian Motion in Glycerol‐Water Mixtures

To evaluate trajectory‐linking performance in physically verifiable systems, we analyze the diffusion of 200 and 30 nm fluorescent beads in 15%–60% glycerol‐water solutions (Figures S8 and S9). The diffusion coefficient (D) in this system can be quantitatively predicted via the Stokes–Einstein equation [52, 53, 54], making it an ideal benchmark for testing whether improved linking quality enhances downstream inference (see the ‘Experimental data’ section in the Methods). As shown in Figure 5a, ReLink consistently produces diffusion coefficient estimates that align closely with theoretical predictions across all glycerol volume fractions, with a clear advantage at 60% glycerol where TrackMate and u‐Track deviate more from theory. In addition, ReLink maintains consistent separation between particle sizes, producing diffusivity ratios between 30 and 200 nm beads closer to the theoretical value (approximately 7.4) than other methods (Figure 5a). All approaches recover anomalous exponents (α) close to 1 (Figure S10), confirming free diffusion in this system. These results support physically consistent diffusion inference with ReLink in an experimentally noisy yet physically well‐defined system.

**FIGURE 5:**
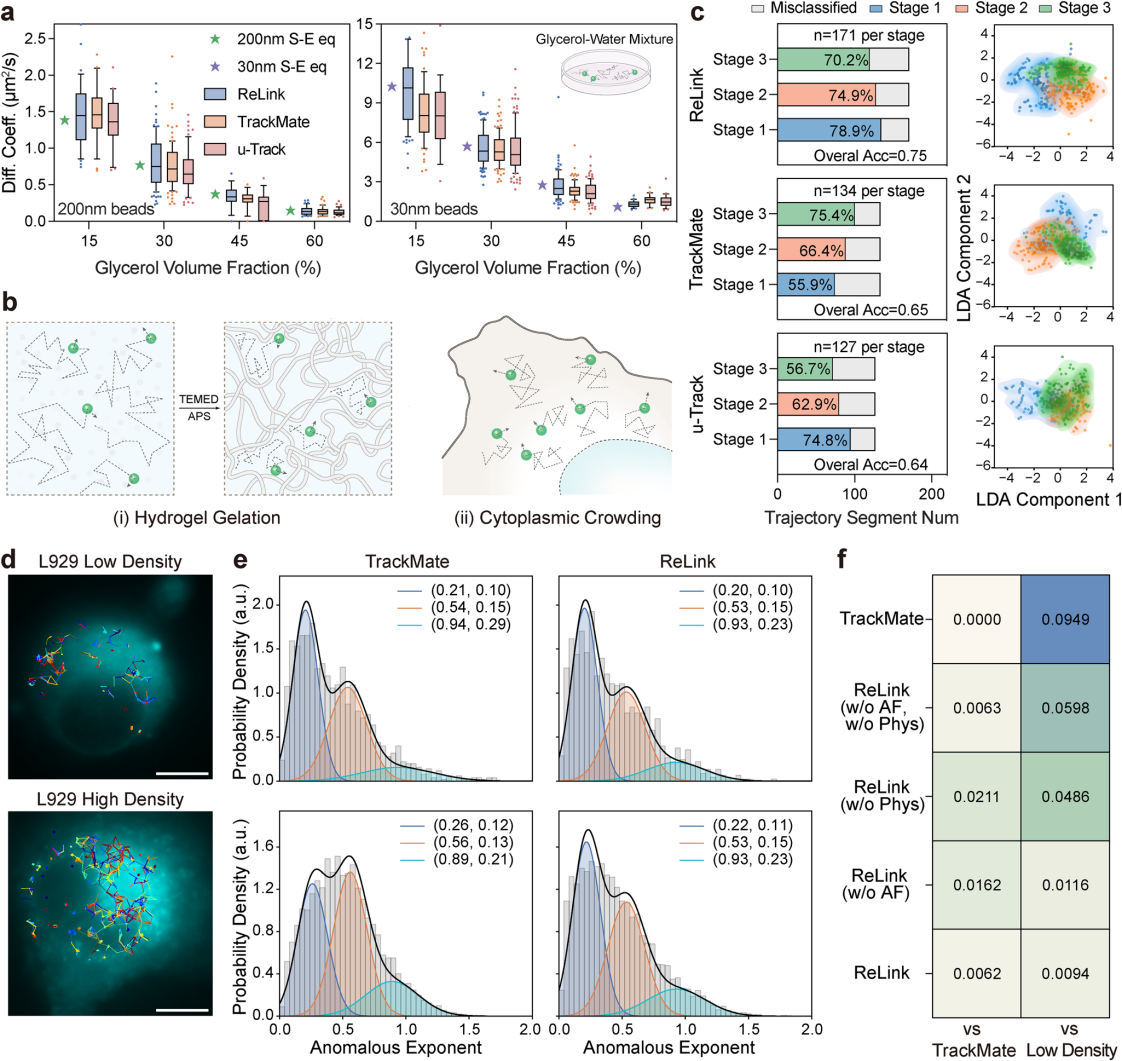
Reliable downstream analysis enabled by ReLink in real‐world SPT experiments. (a) Brownian diffusion of 200 and 30 nm fluorescent beads in glycerol‐water mixtures. Diffusion coefficients estimated from trajectories reconstructed by ReLink, TrackMate, and u‐Track are shown, together with theoretical values computed from the Stokes–Einstein equation (asterisks). The inset depicts beads undergoing Brownian motion in the glycerol‐water mixture. The theoretical diffusivity ratio between 30 and 200 nm beads is 7.4. Measured ratios across glycerol fractions are 7.0, 7.1, 7.5, and 9.9 for ReLink; 5.5, 7.4, 7.5, and 12.5 for TrackMate; 5.9, 7.8, 7.8, and 12.8 for u‐Track. Box plots show the median (center line), interquartile range (box), and the 10th–90th percentiles (whiskers). Points outside this range indicate the tails of the distribution rather than statistical outliers. (b) Schematics of two experimental scenarios for downstream analysis: (i) Probing diffusion‐state evolution during hydrogel gelation, where particle motion becomes increasingly constrained. (ii) Intracellular tracking of quantum dots within L929 cells, illustrating anomalous diffusion in the crowded cytoplasmic environment. (c) Classification of trajectory segments from the three gelation stages. Colored bars denote correctly classified segments for each stage, whereas gray portions denote misclassified segments; stage‐wise and overall accuracies are indicated. The corresponding linear discriminant analysis (LDA) projections of individual trajectory segments are shown on the right. (d) Representative examples of quantum dot motion in L929 cells under low‐density (top) and high‐density (bottom) conditions. Scale bars, 10 µm. (e) Distributions of anomalous exponents (α) and corresponding Gaussian mixture model (GMM) fits for trajectories reconstructed by TrackMate and ReLink under low‐density (top) and high‐density (bottom) conditions. The mean (µ) and standard deviation (σ) of individual GMM components are indicated. (f) Ablation analysis of the physical statistics module (Phys) and the adaptive filtering module (AF), including removal of each module individually and removal of both modules together from the complete ReLink framework. Values denote the symmetric KL divergences between the resulting anomalous‐exponent (α) distributions and the corresponding reference distributions. Lower values indicate greater distributional similarity. The first column compares each low‐density distribution with the low‐density TrackMate distribution, whereas the second compares the high‐density result with the corresponding low‐density result.

#### 2.5.2 Diffusion‐State Evolution During Hydrogel Gelation

During hydrogel gelation, the system gradually transforms from a monomer state into a cross‐linked network, progressively increasing spatial confinement and imposing time‐varying constraints on particle motion (Figure 5b). To capture this evolving complexity, the gelation process is divided into three temporal stages, allowing systematic evaluation of particle dynamics under evolving confinement (see the ‘Experimental data’ section in the Methods). As shown in Figure 5c, trajectories reconstructed by ReLink achieve better three‐state classification accuracy compared with TrackMate or u‐Track (Figure S11). Across all three stages, ReLink recovers more valid trajectories and yields more accurately labeled segments for each stage, resulting in an overall accuracy of 0.75 (vs. 0.65 for TrackMate and 0.64 for u‐Track). The linear discriminant analysis (LDA) visualization further shows that ReLink produces clearly separable clusters corresponding to the three stages, whereas traditional methods exhibit substantial overlap due to linking errors and fragmented trajectories, blurring boundaries between stages. These analyses indicate that fine‐grained motion states that are visually similar yet physically distinct in evolving environments can be more precisely characterized and classified when supported by ReLink's reliable trajectory linking.

#### 2.5.3 Anomalous Diffusion in L929 Cells

Real cellular environments pose challenges beyond those encountered in simulations, including spatial heterogeneity and background fluorescence. To evaluate trajectory linking robustness under these conditions, the L929 mouse fibroblast cell line loaded with quantum dots is used as a representative system exhibiting confined anomalous diffusion (Figure 5b; see the ‘Experimental data’ section in the Methods). We compare low‐ and high‐density recordings, where the intracellular medium experienced by the quantum dots is expected to be similar, so population‐level anomalous diffusion statistics should remain comparable across densities and can serve as an internal consistency check [10, 55, 56]. Representative trajectories reconstructed by ReLink both show restricted diffusion with occasional larger displacements (Figure 5d). Under low‐density conditions where trajectory linking is relatively unambiguous, TrackMate results are used as an approximate reference, showing strongly restricted diffusion with anomalous‐exponent (α) values concentrated around two dominant components (Figure 5e, top left). To quantify the similarity between the estimated α distributions and the corresponding reference distributions, we use the symmetric Kullback‐Leibler (KL) divergence, defined as the average of the two directional KL divergences between a pair of distributions. Trajectories reconstructed by ReLink under the same low‐density condition (Figure 5e, top right) closely match the corresponding TrackMate reference distribution (symmetric KL divergence = 0.006). When challenged with the high‐density data, ReLink remains highly consistent with its low‐density result (symmetric KL divergence = 0.009; Figure 5e, bottom right). In contrast, TrackMate exhibits a pronounced density‐dependent shift (Figure 5e, bottom left), with a substantially larger divergence (0.095, whereas ReLink achieves an approximately 10× lower divergence), biasing α toward higher values and increasing the apparent fraction of near‐Brownian motion. Such deviations are likely attributable to linking errors under high‐density conditions, including trajectory fragmentation and spurious long‐range jumps. Ablation analyses further show that removing either the physical statistics module or the adaptive filtering module increases the distributional deviations (Figure 5f; Figure S12). These results indicate that both modules contribute to maintaining consistent anomalous‐diffusion inference in dense and heterogeneous cellular environments.

## 3 Discussion

Despite notable advances in trajectory‐linking accuracy, existing methods offer limited insight into how links are formed and how reliable individual links are. As a result, researchers often lack effective tools to evaluate, interpret, and selectively retain trustworthy trajectories, particularly in complex and ambiguous experimental settings. To address this gap, we introduce ReLink as a framework that integrates a physics‐informed graph neural network with an adaptive confidence filtering module to retain high‐confidence links across diverse experimental conditions. A key feature of ReLink is its explicit encoding of physical statistical priors during the feature construction stage, capturing essential dynamical characteristics of particle motion by modeling multi‐scale local and global jump‐distance distributions as inductive biases. This physical statistics module facilitates faster and more stable training (Figure S13), leads to more accurate linking prediction and provides a robust foundation for subsequent confidence‐based filtering. The effectiveness of this design is validated through extensive simulation studies spanning variations in particle diffusion models, video length, particle density, and signal‐to‐noise ratio that induce false detections and localization errors. Furthermore, the empirically observed bimodal structure of confidence distributions rationalizes the use of the valley between modes as an adaptive and balanced threshold, a choice supported by confidence‐distribution analyses and mean‐confidence diagrams. Beyond simulations, the impact of our accurate and reliable linking is demonstrated on established benchmarks as well as in experiments of increasing environmental complexity, where improved trajectory reliability substantially simplifies and enhances downstream SPT analyses in realistic biological systems.

ReLink exhibits strong robustness and generalization capability across motion regimes and experimental domains, although the network training was primarily performed on FBM‐based particle trajectories. In AnDi 1 evaluations, ReLink can accurately link trajectories generated by annealed transient time motion (ATTM), continuous‐time random walk (CTRW), and Lévy walk (LW) models, excelling in both diffusion‐type classification and anomalous‐exponent estimation. While performance becomes slightly worse on scaled Brownian motion (SBM), which is closely related to but distinct from FBM [57, 58], this suggests that augmenting the training procedure with broader motion models could further improve the framework's generalization. Notably, despite the absence of splitting events in the training data and the mismatch between simulated particle motion and cellular dynamics, ReLink successfully reconstructs HeLa trajectories and detects division events, highlighting its strong cross‐domain generalization. In real‐world cellular environments characterized by heterogeneity and crowding, FBM‐based training already provides a reliable default. Nevertheless, expanding the training set to include more diverse motion types or adopting data‐augmentation strategies like trajectory mix‐up [59] may further enhance universality. Moreover, experiments on the Cell Tracking Challenge reveal the data efficiency of our framework, as a model trained using labeled trajectories from only a single HeLa video is sufficient to yield high‐quality linking on MuSC data without target‐specific fine‐tuning, highlighting effective transfer across cell types. We hypothesize that the physical statistics module also serves as a regularizer by encouraging the network to learn representations consistent with motion governed by stochastic differential equations (SDEs), thereby preventing overfitting to source‐domain characteristics. In future work, we may explore more comprehensive explicit or implicit modeling of particle‐motion SDEs or PDEs [60, 61], for example by incorporating SDE‐based diffusion models [62] or autoencoders to generate particle motions [63]. Such extensions could further improve linking accuracy, zero‐shot capability, and generalization, and may ultimately enable self‐supervised or unsupervised trajectory linking.

An additional consideration concerns the application of the confidence‐based adaptive threshold filtering strategy. The current adaptive filtering module relies on the assumption of bimodal confidence distributions, while manual threshold selection is supported as a fallback where careful manual tuning may yield higher‐quality trajectories in specific scenarios. Results on the AnDi 2 Benchmark illuminate the inherent trade‐off in thresholding: higher thresholds favor ensemble statistics by retaining higher‐quality predictions, whereas lower thresholds preserve a larger number of trajectories for single‐trajectory analysis. The adaptive threshold provides a balanced compromise that benefits both perspectives. Nonetheless, confidence distributions may deviate from bimodality in certain cases (becoming unimodal or multimodal), where the current adaptive strategy may be suboptimal or fail altogether. In such situations, manual thresholding or even bypassing confidence‐based filtering may be appropriate. Future extensions of ReLink may further benefit from incorporating spectral information of particles, enabling joint exploitation of motion and spectral cues for more reliable trajectory reconstruction [64, 65, 66].

Overall, ReLink provides a general and flexible solution for trajectory linking in single‐particle tracking. By integrating physically grounded statistical representations with confidence‐aware adaptive filtering, ReLink enables reliable and interpretable trajectory reconstruction under challenging experimental conditions. More broadly, ReLink offers a principled basis for associating stochastic motion, supporting robust downstream analysis in complex biological and physical systems.

## 4 Methods

### 4.1 Candidate Graph Construction

Each graph G=(V,E) is constructed from single‐particle detections in one input sequence and contains both localization information and physical statistics computed from the same sequence. The physical statistics are derived from local and global jump‐distance histograms calculated as described below, which summarize observed displacement patterns over multiple temporal intervals and spatial ranges and provide motion features relevant to candidate links.

#### 4.1.1 Nodes

Each node i∈V corresponds to a detection (**x**_*i*_, *t_i_*), where $x_{i}=\left(x_{i,1},x_{i,2}\right)\in R^{2}$ denotes the 2D spatial coordinates and *t_i_* denotes the frame index. Spatial coordinates can be optionally normalized to [0, 1], and the choice of normalization leads to only minor quantitative differences. Ground‐truth trajectory identities (when available) are retained solely for supervision.

#### 4.1.2 Edges

Candidate edges are constructed between nodes *i* (**x**_*i*_,*t_i_*) and *j* (**x**_*j*_,*t_j_*) when their spatial distance does not exceed *r*_max_ and their temporal separation is within Δ*t*_max_ which is set to 2 during training and 1 during inference:

(1) $$E=\left\{\left(i,j\right)|1\leq t_{j}-t_{i}\leq \Delta t_{\max},\;\;∥x_{j}-x_{i}∥\leq r_{\max}\right\}.$$

The attributes of each candidate edge include the per‐axis displacement between the two endpoints, the time difference, the spatial distance, and the speed. For supervised training, we assign a binary label $y_{ij}\in \left\{0,1\right\}$, where *y_ij_* =  1 denotes a true link (the two detections belong to the same ground‐truth trajectory) and *y_ij_* =  0 denotes a non‐link.

#### 4.1.3 Local Statistical Features

For each node i∈V, we compute jump distances to all neighboring nodes within a local radius *r*_local_ (typically chosen to cover no more than 25% of the field of view width) at time lag Δt∈{1,…,T}, where *T* denotes the number of temporal lags used to construct the physical‐statistics features. In this work, we set *T*  =  3. The local spatial neighborhood is defined as

(2) $$N_{i}\;\left(r_{\mathrm{local}},\Delta t\right)=\left\{j\in V|\;t_{j}=t_{i}+\Delta t,\;∥x_{j}-x_{i}∥\leq r_{\mathrm{local}}\right\}\;.$$

The set of all jump distances associated with node *i* is

(3) $$D_{i}^{\Delta t}=\left\{d_{i,j}^{\Delta t}=∥x_{j}-x_{i}∥\;|\;j\in N_{i}\left(r_{\mathrm{local}},\Delta t\right)\right\}\;.$$

For each Δ*t*, we compute (i) the mean jump distance

(4) $$\mu_{i}^{\Delta t}=\frac{1}{|D_{i}^{\Delta t}|}\sum_{d\in D_{i}^{\Delta t}}d,$$

and (ii) a *B*‐bin normalized histogram of the local jump‐distance distribution $h_{i,\mathrm{local}}^{\Delta t}\in R^{B}$, satisfying $\sum_{b=1}^{B}\;h_{i,\mathrm{local},\;b}^{\Delta t}=1.$ Collecting the mean jump distance over all nodes and Δ*t* forms the local mean feature $M_{\mathrm{local}}\in R^{|V|\times T\times 1}$ and collecting all local histograms $h_{i,\mathrm{local}}^{\Delta t}$ yields the local histogram feature $H_{\mathrm{local}}\in R^{|V|\times T\times B}$.

#### 4.1.4 Global Statistical Features

Similar to the local statistics, we compute the set of global jump distances at each lag Δt∈{1,2,3} by aggregating over all eligible node pairs within a global spatial range *r*_global_ (typically chosen to span the full field of view):

(5) $$\begin{array}{c} D_{\mathrm{global}}^{\Delta t}=\left\{∥x_{j}-x_{i}∥\;|i,j\in V,\;t_{j}=t_{i}+\Delta t,\;∥x_{j}-x_{i}∥\leq r_{\mathrm{global}}\right\}\;. \end{array}$$

The *B*‐bin normalized histogram $h_{\mathrm{global}}^{\Delta t}\in R^{B}$ is computed and broadcast across nodes, yielding the global histogram feature $H_{\mathrm{global}}\in R^{|V|\times T\times B}$ where $h_{i,\mathrm{global}}^{\Delta t}=h_{\mathrm{global}}^{\Delta t}$ for all *i*.

### 4.2 Physics‐Informed GNN

The network takes a pre‐constructed candidate graph (nodes and edge attributes) as input (Figure S14). Raw node features are embedded by a node encoder (a multilayer perceptron + LayerNorm) into a hidden space (hidden dimension). In parallel, edge attributes are embedded by an edge encoder (a multilayer perceptron + LayerNorm) into the same hidden dimension, yielding the initial edge embedding **e**_*ij*_. We then apply a graph attention network (GAT) [67] layer with edge attributes to perform local aggregation of node features, while a Transformer [41] encoder learns global long‐range temporal dependencies. The resulting local and global node features are fused via a linear projection, producing node representations **h**_*i*_.

We next incorporate the jump‐distance statistics as physical priors into the node and edge representations after the initial graph features are obtained and before candidate‐edge classification. Specifically, we first compute the discrete cumulative distribution function (CDF) $F_{\mathrm{global}}^{\Delta t}$ by cumulatively summing the normalized global histogram $h_{\mathrm{global}}^{\Delta t}$ over bins. For each node *i*, we map its local mean jump distance $\mu_{i}^{\Delta t}=M_{\mathrm{local}}\;\left[i,\Delta t\right]$ to a histogram bin using fixed bin boundaries (with out‐of‐range indices clamped) and retrieve the corresponding CDF value as:

(6) $$s_{i}^{\Delta t}=F_{\mathrm{global}}^{\Delta t}\;\left(\mu_{i}^{\Delta t}\right).$$

We then stack these lag‐specific scores into a vector $S_{i}={\left[s_{i}^{\Delta t}\right]}_{\Delta t=1}^{T}$ with *T*  =  3. The stacked scores across lags are mapped by a Score Net, implemented as a two‐layer multilayer perceptron (MLP_score_), and concatenated to the node feature:

(7) $$\phi_{i}=\mathrm{ML}P_{\mathrm{score}}\left(S_{i}\right),\;$$

(8) $$h_{i}^{\prime }=\mathrm{Proj}\left(\mathrm{concat}\left(h_{i},\phi_{i}\right)\right).$$

To extract the correspondence between local and global jump‐distance statistics, we modulate edge embeddings using a Histogram Net (Figure S14). For each lag Δt∈{1,2,3}, we implement Feature‐wise Linear Modulation (FiLM) [40] using source‐node local and global jump‐distance histograms to obtain modulation coefficients:

(9) $$\left(\gamma_{i,\mathrm{local}}^{\Delta t},\beta_{i,\mathrm{local}}^{\Delta t}\right)={\mathrm{MLP}}_{\mathrm{local}}^{\Delta t}\left(h_{i,\mathrm{local}}^{\Delta t}\right),\;$$

(10) $$\left(\gamma_{i,\mathrm{global}}^{\Delta t},\beta_{i,\mathrm{global}}^{\Delta t}\right)={\mathrm{MLP}}_{\mathrm{global}}^{\Delta t}\left(h_{i,\;\mathrm{global}}^{\Delta t}\right),$$

where $h_{i,\mathrm{local}}^{\Delta t}=H_{\mathrm{local}}\;\left[i,\Delta t\right]$ denotes the local histogram and $h_{i,\mathrm{global}}^{\Delta t}=H_{\mathrm{global}}\;\left[i,\Delta t\right]$ denotes the global histogram. The resulting coefficients are shared across all outgoing edges from node *i* at the same lag Δ*t*. Given an edge embedding **e**_*ij*_, FiLM modulation is applied as

(11) $${\hat{e}}_{\mathrm{ij},\mathrm{local}}^{\Delta t}=\gamma_{i,\mathrm{local}}^{\Delta t}⊙e_{\mathrm{ij}}+\beta_{i,\mathrm{local}}^{\Delta t},$$

(12) $${\hat{e}}_{ij,\mathrm{global}}^{\Delta t}=\gamma_{i,\mathrm{global}}^{\Delta t}\;\;⊙e_{ij}+\beta_{i,\mathrm{global}}^{\Delta t},$$

where ⊙ denotes the element‐wise multiplication. We concatenate the FiLM‐modulated local and global edge features, project them into an embedding, and stack the resulting embeddings across Δ*t* to form a token sequence:

(13) $$U_{ij}={\left[\mathrm{Proj}\left(\left[{\hat{e}}_{ij,\mathrm{local}}^{\Delta t};{\hat{e}}_{ij,\mathrm{global}}^{\Delta t}\right]\right)\right]}_{\Delta t=1}^{T}\;,$$

A multi‐head attention (MHA) module is then applied to fuse information across lags in a single‐query manner: the query is the mean token over lags, while keys and values are the lag tokens. The attention output is added to the original edge embedding via a residual connection, yielding $e_{ij}^{\mathrm{fused}}$:

(14) $$e_{ij}^{\mathrm{fused}}=\mathrm{MHA}\left(\mathrm{MeanPool}\left(U_{ij}\right),U_{ij},U_{ij}\right)+e_{ij}.$$

Finally, we apply another GAT layer (GATConv) to propagate the fused edge features (now containing local and global statistical priors) back to the node features:

(15)

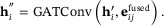




After incorporating physical statistics priors, the network predicts a logit *z_ij_* for each candidate edge (*i*, *j*) by concatenating the source‐node features, target‐node features, and the fused edge features:

(16)

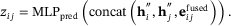




The corresponding probability *p_ij_* =  σ(*z_ij_*), where σ (*u*) =  1/(1 + exp (− *u*)) denotes the sigmoid function, indicates whether the candidate edge is positive. All candidate edges and their probabilities are subsequently passed to the post‐processing module for trajectory reconstruction.

### 4.3 Post‐Processing for Trajectory Reconstruction

The network outputs only the probability *p_ij_* for each candidate edge (*i*, *j*). Trajectories are reconstructed via post‐processing that combines frame‐to‐frame assignment and gap closing. Post‐processing constraints are specified on a per‐dataset basis to account for differences in imaging conditions, and are kept identical across all applicable linking methods within each dataset to ensure a fair comparison (Table S8). We first perform frame‐to‐frame association using the Hungarian algorithm (in our experiments, between consecutive frames), with linking cost 1 −  *p_ij_* . This defines the Hungarian cost matrix, enabling a globally optimal matching that minimizes the overall linking cost. The same formulation readily extends to association over larger frame gaps when such candidate edges are provided. A greedy strategy with a binary threshold (e.g., *p_ij_* > 0.5) may also be used, but typically produces inferior results. After frame‐to‐frame association, we perform gap closing under a temporal constraint Δ *t_ij_* = *t_j_*  − *t_i_* ≤ *T*_gap_ and a spatial constraint *d_ij_* = ‖**x**_*j*_ − **x**_*i*_‖  ≤ *d*_gap_. For any pair of detections satisfying these constraints, we again apply the Hungarian algorithm to obtain the minimal‐cost gap‐closing configuration, where each entry of the gap‐closing cost matrix is computed as *d_ij_*/*d*_gap_ + Δ*t_ij_*/*T*_gap_. In most scenarios, these two steps are sufficient to reconstruct stable trajectories.

For the Cell Tracking Challenge dataset, we additionally incorporate cell‐division (split) detection (see Note S1). Given a set of reconstructed trajectories, if the start node of one trajectory has a candidate connection probability (*p*_2_) to a middle node of another trajectory, and that middle node already has an outgoing link with probability (*p*_1_), a split event is detected when (*p*_1_ − *p*_2_)/*p*_1_ ≤ τ_split_ where τ_split_ is a split threshold. The threshold is empirically selected for each dataset (0.01 for HeLa, 0.3 for MuSC). Rather than using a hard threshold such as (*p*_2_ > 0.5), this criterion directly measures the deviation between the best alternative link and the existing link, providing a more intuitive signal for division events.

### 4.4 Confidence Estimation and Adaptive Threshold Filtering

To quantify link‐level confidence, we adopt Monte Carlo dropout [42] as an approximate Bayesian inference scheme. Let *f*(·; θ) denote the trained trajectory‐linking network, where θ represents the learned network parameters. Keeping dropout active at inference induces a variational distribution *q*_θ_(**w**) over stochastic network realizations **w** (via independently sampled Bernoulli dropout masks). For each candidate edge e=(i,j)∈E, we perform *N*  =  50 stochastic forward passes with $w^{\left(n\right)}∼q_{\theta }\left(w\right)$ and obtain the logit of the *n*‐th pass:

(17) $$z_{e}^{\left(n\right)}=f\left(e;\;w^{\left(n\right)}\right).$$

The corresponding probability is computed as

(18) $$p_{e}^{\left(n\right)}=\sigma \left(z_{e}^{\left(n\right)}\right).\;$$

The Monte Carlo approximation of the predictive distribution is

(19) $${\hat{p}}_{e}\approx \frac{1}{N}\sum_{n=1}^{N}p_{e}^{\left(n\right)}.$$

We treat ${\hat{p}}_{e}$ as the parameter of a Bernoulli distribution and compute the entropy to measure total uncertainty:

(20) $$H\;\left({\hat{p}}_{e}\right)=-{\hat{p}}_{e}\log_{2}{\hat{p}}_{e}-\left(1-{\hat{p}}_{e}\right)\log_{2}\left(1-{\hat{p}}_{e}\right),$$

(21) $$c_{e}=1\;-\;H\left({\hat{p}}_{e}\right),$$

where *c_e_* serves as a confidence score (larger values indicate more confident predictions).

Empirically, the confidence scores over all candidate edges are approximately bimodal: the right peak corresponds to high‐confidence edges with higher accuracy, and the left peak corresponds to low‐confidence edges with lower accuracy. We therefore identify the density valley between two dominant peaks as an adaptive optimal (or balanced) threshold. Specifically, to avoid the sensitivity to the size of histogram bins, we fit the distribution of confidence via kernel density estimation (KDE) [43]:

(22) $$\hat{f}\left(c\right)=\frac{1}{\left|E\right|\sigma_{\mathrm{bw}}}\sum_{e\in E}K\left(\frac{c-c_{e}}{\sigma_{\mathrm{bw}}}\right),$$

where *K*(·) is a Gaussian kernel function. The bandwidth is determined separately for each input sequence using Scott's rule, $\sigma_{\mathrm{bw}}=sd_{c}\;{\left|E\right|}^{-1/5}$, where sd_*c*_ is the sample standard deviation of the edge confidence scores. We locate the two main peaks (*c_L_*, *c_R_*) by solving for the zeros of the derivative of this KDE curve and define the adaptive threshold as the minimum between them:

(23) $$\tau^{*}=\arg\min_{c\in \left(c_{L},c_{R}\right)}\;\;\hat{f}\left(c\right).$$

At inference, candidate edges with *c_e_* < τ* are discarded. By applying this adaptive filtering module, only high‐confidence candidate edges are propagated to the post‐processing stage, enabling the construction of reliable trajectories. The corresponding computation process is shown in Algorithm S1.

### 4.5 Model Training and Inference

Our task is to train the network to predict whether a candidate edge (*i*,  *j*) corresponds to a true link between two detections. The loss function is therefore a binary cross‐entropy (BCE). For each candidate edge (i,j)∈E, the network outputs a logit *z_ij_* and the corresponding linking probability *p_ij_* =  σ(*z_ij_*). The BCE loss over all candidate edges is

(24) $$L=\frac{1}{\left|E\right|}\;\sum_{\left(i,j\right)\in E}\left[-y_{ij}\log p_{ij}-\left(1-y_{ij}\right)\log\left(1-p_{ij}\right)\right],$$

where $y_{ij}\in \left\{0,1\right\}$ denotes the ground‐truth label for edge (*i*, *j*). We use AdamW as the optimizer with an initial learning rate of 10^−3^, which is decayed over epochs. The dropout rate for the network is 0.3. The FiLM layers are initialized to approximate an identity mapping to stabilize training. All architectural choices and training hyperparameters (e.g., hidden feature dimensions and number of layers) are fixed across datasets and summarized in Table S9. The local and global jump‐distance histograms are calculated directly from the localization coordinates of each input sequence rather than learned during training. During inference, the histograms are recalculated for each new sequence, while the trained network parameters remain fixed.

### 4.6 Metrics for Evaluating Trajectory Linking

To quantitatively evaluate linking performance, we adopt metrics at two complementary levels: (i) Edge‐level metrics (Edge F1 Score) characterize whether candidate edges are correctly classified. (ii) Trajectory‐level metrics quantify how well the final reconstructed trajectories match the GT trajectories. Following standard multi‐object tracking (MOT) [68] practices, we compute Multiple Object Tracking Accuracy (MOTA). Predicted detections are first matched to ground‐truth detections within a Euclidean distance of 1 pixel, and the resulting correspondences are used to determine whether predicted edges match ground‐truth edges.

#### 4.6.1 Edge F1 Score

This score measures the harmonic mean of precision and recall for edge classification:

(25) $$\mathrm{Precision}\;=\frac{TP_{\mathrm{edge}}}{TP_{\mathrm{edge}}+FP_{\mathrm{edge}}}\;,$$

(26) $$\mathrm{Recall}\;=\frac{TP_{\mathrm{edge}}}{TP_{\mathrm{edge}}+FN_{\mathrm{edge}}}\;,$$

(27) $$\mathrm{Edge}\;F1=2\times \frac{\mathrm{Precision}\times \mathrm{Recall}}{\mathrm{Precision}+\mathrm{Recall}},$$

where TP_edge_, FP_edge_ and FN_edge_ denote true positive, false positive and false negative edge predictions.

#### 4.6.2 MOTA

This representative MOT metric combines false negatives, false positives and identity‐switch errors into a single score:

(28) $$\mathrm{MOTA}\;=1-\frac{FN_{\mathrm{node}}+FP_{\mathrm{node}}+\mathrm{IDSW}}{N_{\mathrm{GT}}},\;$$

where FN_node_, FP_node_, IDSW and N_GT_ denote missed detections, spurious detections, the number of identity switches and total number of ground‐truth detections. Higher MOTA indicates more consistent and identity‐stable trajectory reconstruction.

### 4.7 Simulations

To systematically assess linking accuracy under diverse physical and imaging conditions, we constructed a comprehensive simulation benchmark based on the *andi‐datasets* framework [44]. The benchmark spanned a wide range of trajectory lengths, particle densities, state‐switching behaviors, and SNR levels, enabling us to probe both the upper limits of linking performance (ideal localization) and robustness under realistic noise. Detailed configurations for generating simulated datasets are provided in Note S2, and settings for public benchmark datasets are summarized in Note S3.

Synthetic trajectories were generated using FBM‐based models to emulate heterogeneous diffusion, transient particle association and spatial restriction, enabling a controlled evaluation of ReLink under complex and non‐stationary dynamics [69].

#### 4.7.1 FBM

Fractional Brownian Motion (FBM) [70] is a zero‐mean Gaussian process *B_H_* (*t*) characterized with the Hurst exponent H∈(0,1) and covariance function

(29) $$E\left[B_{H}\left(t\right)B_{H}\left(s\right)\right]=\frac{1}{2}\left({\left|t\right|}^{2H}+{\left|s\right|}^{2H}-{\left|t-s\right|}^{2H}\right),$$

where E[·] denotes the expected value. The particle trajectory in *d*‐dimensional space is defined as

(30) $$x\left(t\right)=x_{0}+\sqrt{2D}B_{H}\left(t\right),$$

where **B**_*H*_(*t*) denotes a *d*‐dimensional FBM with independent components, *D* is the generalized diffusion coefficient, and *H* is the Hurst exponent, which is related to the anomalous diffusion exponent α. The corresponding mean squared displacement at time lag τ is given by

(31) $$E\left[∥x\left(t+\tau \right)-x\left(t\right){∥}^{2}\right]\propto \tau^{\alpha },\;\alpha =2H.$$

#### 4.7.2 Multi‐State Model

In the multi‐state setting, a particle switches between *K* latent dynamical states s(t)∈{1,2,…,K}, where each state *k* is associated with parameters (*D_k_*,*H_k_*). The dynamics are governed by

(32) $$x\left(t+\Delta t\right)=x\left(t\right)+\sqrt{2D_{s\left(t\right)}}\left[B_{H_{s\left(t\right)}}\left(t+\Delta t\right)-B_{H_{s\left(t\right)}}\left(t\right)\right],$$

where Δ*t* is the sampling interval. The state transitions follow a Markov process:

(33) $$\mathrm{Pr}\left(s\left(t+\Delta t\right)=j|s\left(t\right)=i\right)=\pi_{\mathrm{ij}},\;\;\;\;\sum_{j=1}^{K}\pi_{\mathrm{ij}}=1,\forall i.$$

where π_*ij*_ is the Markov transition probability from state *i* to *j*. This model mimics heterogeneous environments with time‐varying transport properties.

#### 4.7.3 Dimerization Model

The dimerization model extends the FBM dynamics by allowing particles to switch between monomeric and dimeric states, which are characterized by distinct diffusion parameters (*D*,  α). Two particles separated by *d* < 2*r* bind with probability *P_b_*, upon which both particles switch to the dimeric state and undergo identical displacements. The dimer dissociates with probability *P_u_* at each time step, after which the particles return to their original monomeric diffusion parameters.

#### 4.7.4 Confinement Model

The confinement model describes particle motion inside and outside *N_c_* randomly distributed non‐overlapping circular compartments of radius *r_c_*. The two regions are assigned distinct diffusion parameters (*D*,  α). A particle reaching a compartment boundary from outside enters the compartment, whereas a particle reaching the boundary from inside exits with probability *P*_exit_ and is otherwise reflected back into the compartment. Thus, transitions between the free and confined diffusion states are determined by particle position and boundary crossing.

#### 4.7.5 Immobilization Model

Temporary immobilization is modeled by introducing stationary traps into the diffusion environment. A particle entering a trap region binds with probability *P_b_* upon which its diffusion parameters are set to *D*  =  0 and α  =  0. At each subsequent time step, the immobilized particle is released with probability *P_u_* and resumes its original diffusion dynamics.

### 4.8 Experimental Data

In our work, we analyzed three types of experimental SPT data: Brownian motion in glycerol‐water mixtures, diffusion‐state evolution during hydrogel gelation, and anomalous diffusion in L929 cells. All experimental SPT data were acquired using an EMCCD camera (Andor, iXon Life 888 EMCCD) on our home‐built microscope with a 1.49 NA objective (Nikon, Apochromat TIRF 100XC Oil) and excitation lasers at 405, 561, and 637 nm (see Note S4 and Figure S15 for details).

For the bead diffusion experiments in glycerol‐water mixtures, fluorescent beads with nominal diameters of approximately 200 nm (Thermofisher, F8806) and 30 nm (Thermofisher, F8782) were diluted 1:4000 and dispersed in glycerol‐water mixtures with glycerol volume fractions of 15%, 30%, 45%, and 60%. To assess diffusion in a physically well‐defined system, 200‐frame image sequences were recorded for each condition. Particle localizations were obtained using TrackMate (DoG), after which trajectories were reconstructed using ReLink, TrackMate, and u‐Track, respectively. The resulting trajectories were subsequently analyzed using U‐LFormer [45] to estimate the diffusion coefficient (D) and the anomalous exponent (α). In this system, the diffusion coefficient can be predicted by the Stokes–Einstein equation [53, 54]:

$$D=\frac{k_{B}T}{6\pi \eta r}\;,$$

where *k_B_* is the Boltzmann constant, *T* is the absolute temperature, *η* is the dynamic viscosity of the solution, and *r* is the particle radius. This analytical expression enables quantitative comparison between experimentally inferred diffusion coefficients and theoretical expectations.

Hydrogel gelation experiments followed the thermal curing protocol in Note S5. To quantify diffusion‐state evolution during gelation, we acquired 300‐frame image sequences with 100 frames for each stage. After TrackMate (DoG) localization, trajectories were reconstructed using different linking algorithms, sliced into equal‐length segments with a sliding window and classified into three diffusion states using the Diffusional Fingerprinting method [37]. All reconstructed trajectories were segmented and the segments were randomly subsampled to balance the three stages. Classification performance was evaluated using fivefold cross‐validation, yielding a balanced and fair comparison across methods.

For the anomalous diffusion in L929 experiments, cell culture and electroporation‐based delivery of quantum dots followed the protocol in Note S5. Quantum dots were imaged for 200 frames at two localization densities: low (23 cells; around 30 localizations per frame per cell) and high (21 cells; around 150 localizations per frame per cell). Following localization with TrackMate (DoG), trajectories were reconstructed using ReLink and TrackMate. Anomalous exponents (α) were estimated using U‐LFormer, and their distributions were fitted with Gaussian mixture models (GMMs), with the number of components selected by minimizing the Bayesian information criterion (BIC).

## Author Contributions

Y.J., X.F., and Y.Z. initiated the project. Y.J., X.F., and H. Wei conducted the experiments. H. Wei contributed to sample preparation. X.F. contributed to building the optical system. Y.J., X.F., H. Wei, H.S., and H. Wang analyzed the results. Y.J. and X.F. wrote the manuscript with feedback from all authors. M.Z., S.H., and Y.Z. supervised the project. Y. Z. critically reviewed, edited, and approved the manuscript.

## Conflicts of Interest

The authors declare no conflicts of interest.

## Code Availability

The code used in this study is publicly available on Zenodo at https://doi.org/10.5281/zenodo.21802802.

## Supporting information




**Supporting File**: advs78541‐sup‐0001‐SuppMat.pdf.

## Data Availability

The data that support the findings of this study are available on Zenodo at https://doi.org/10.5281/zenodo.21802802.

## References

[1] H. Shen , L. J. Tauzin , R. Baiyasi , et al., “Single Particle Tracking: From Theory to Biophysical Applications,” Chemical Reviews 117, no. 11 (2017): 7331–7376, 10.1021/acs.chemrev.6b00815. 28520419

[2] J. Elf and I. Barkefors , “Single‐Molecule Kinetics in Living Cells,” Annual Review of Biochemistry 88, no. 1 (2019): 635–659, 10.1146/annurev-biochem-013118-110801. 30359080

[3] F. Simon , L. E. Weiss , and S. van Teeffelen , “A Guide to Single‐Particle Tracking,” Nature Reviews Methods Primers 4, no. 1 (2024): 66, 10.1038/s43586-024-00341-3.

[4] R. Metzler and J. Klafter , “The Random Walk's Guide to Anomalous Diffusion: A Fractional Dynamics Approach,” Physics Reports 339, no. 1 (2000): 1–77, 10.1016/S0370-1573(00)00070-3.

[5] C. Manzo and M. F. Garcia‐Parajo , “A Review of Progress in Single Particle Tracking: From Methods to Biophysical Insights,” Reports on Progress in Physics 78, no. 12 (2015): 124601, 10.1088/0034-4885/78/12/124601. 26511974

[6] D. Magde , E. Elson , and W. W. Webb , “Thermodynamic Fluctuations in a Reacting System—Measurement by Fluorescence Correlation Spectroscopy,” Physical Review Letters 29, no. 11 (1972): 705–708, 10.1103/PhysRevLett.29.705.

[7] D. Han , N. Korabel , R. Chen , et al., “Deciphering Anomalous Heterogeneous Intracellular Transport With Neural Networks,” eLife 9 (2020): 52224, 10.7554/eLife.52224. PMC7141808 32207687

[8] T. Deguchi , M. K. Iwanski , E.‐M. Schentarra , et al., “Direct Observation of Motor Protein Stepping in Living Cells Using MINFLUX,” Science 379, no. 6636 (2023): 1010–1015, 10.1126/science.ade2676. PMC7614483 36893247

[9] S. Scott , M. Weiss , C. Selhuber‐Unkel , et al., “Extracting, Quantifying, and Comparing Dynamical and Biomechanical Properties of Living Matter Through Single Particle Tracking,” Physical Chemistry Chemical Physics 25, no. 3 (2023): 1513–1537, 10.1039/d2cp01384c. 36546878

[10] M. U. Zahid , L. Ma , S. J. Lim , and A. M. Smith , “Single Quantum Dot Tracking Reveals the Impact of Nanoparticle Surface on Intracellular State,” Nature Communications 9, no. 1 (2018): 1830, 10.1038/s41467-018-04185-w. PMC5940692 29739927

[11] L. Xiang , K. Chen , R. Yan , W. Li , and K. Xu , “Single‐Molecule Displacement Mapping Unveils Nanoscale Heterogeneities in Intracellular Diffusivity,” Nature Methods 17, no. 5 (2020): 524–530, 10.1038/s41592-020-0793-0. PMC7205592 32203387

[12] H. Sha , Y. Wu , Y. Zhang , et al., “Single Molecule Spectrum Dynamics Imaging With 3D Target‐Locking Tracking,” Nature Communications 16, no. 1 (2025): 8686, 10.1038/s41467-025-63787-3. PMC12485062 41027930

[13] C. S. Peng , Y. Zhang , Q. Liu , et al., “Nanometer‐Resolution Tracking of Single Cargo Reveals Dynein Motor Mechanisms,” Nature Chemical Biology 21, no. 5 (2025): 648–656, 10.1038/s41589-024-01694-2. PMC11785820 39090313

[14] J. S. H. Danial and D. Klenerman , “Single Molecule Imaging of Protein Aggregation in Dementia: Methods, Insights and Prospects,” Neurobiology of Disease 153 (2021): 105327, 10.1016/j.nbd.2021.105327. PMC8039184 33705938

[15] J. Yan , L. Cheng , Y. Li , R. Wang , and J. Wang , “Advancements in Single‐Molecule Fluorescence Detection Techniques and Their Expansive Applications in Drug Discovery and Neuroscience,” Biosensors 15, no. 5 (2025): 283, 10.3390/bios15050283. PMC12109833 40422023

[16] S.‐L. Liu , Z.‐G. Wang , H.‐Y. Xie , A.‐A. Liu , D. C. Lamb , and D.‐W. Pang , “Single‐Virus Tracking: From Imaging Methodologies to Virological Applications,” Chemical Reviews 120, no. 3 (2020): 1936–1979, 10.1021/acs.chemrev.9b00692. PMC7075663 31951121

[17] H.‐Y. Liu , Z.‐G. Wang , S.‐L. Liu , and D.‐W. Pang , “Single‐Virus Tracking With Quantum Dots in Live Cells,” Nature Protocols 18, no. 2 (2023): 458–489, 10.1038/s41596-022-00775-7. 36451053

[18] C. S. Spagnolo and S. Luin , “Trajectory Analysis in Single‐Particle Tracking: From Mean Squared Displacement to Machine Learning Approaches,” International Journal of Molecular Sciences 25, no. 16 (2024): 8660, 10.3390/ijms25168660. PMC11354962 39201346

[19] G. Muñoz‐Gil , H. Bachimanchi , J. Pineda , et al., “Quantitative Evaluation of Methods to Analyze Motion Changes in Single‐Particle Experiments,” Nature Communications 16, no. 1 (2025): 6749, 10.1038/s41467-025-61949-x. PMC12283970 40695819

[20] K. J. A. Martens , B. Turkowyd , J. Hohlbein , and U. Endesfelder , “Temporal Analysis of Relative Distances (TARDIS) Is a Robust, Parameter‐Free Alternative to Single‐Particle Tracking,” Nature Methods 21, no. 6 (2024): 1074–1081, 10.1038/s41592-023-02149-7. 38225387

[21] Y. T. Fukai and K. Kawaguchi , “LapTrack: Linear Assignment Particle Tracking With Tunable Metrics,” Bioinformatics 39, no. 1 (2023): btac799, 10.1093/bioinformatics/btac799. PMC9825786 36495181

[22] J.‐Y. Tinevez , N. Perry , J. Schindelin , et al., “TrackMate: An Open and Extensible Platform for Single‐Particle Tracking,” Methods 115 (2017): 80–90, 10.1016/j.ymeth.2016.09.016. 27713081

[23] K. Jaqaman , D. Loerke , M. Mettlen , et al., “Robust Single‐Particle Tracking in Live‐Cell Time‐Lapse Sequences,” Nature Methods 5, no. 8 (2008): 695–702, 10.1038/nmeth.1237. PMC2747604 18641657

[24] N. Chenouard , I. Smal , F. de Chaumont , et al., “Objective Comparison of Particle Tracking Methods,” Nature Methods 11, no. 3 (2014): 281–289, 10.1038/nmeth.2808. PMC4131736 24441936

[25] R. E. Kalman , “A New Approach to Linear Filtering and Prediction Problems,” Journal of Basic Engineering 82, no. 1 (1960): 35–45, 10.1115/1.3662552.

[26] D. Reid , “An Algorithm for Tracking Multiple Targets,” IEEE Transactions on Automatic Control 24, no. 6 (1979): 843–854, 10.1109/TAC.1979.1102177.

[27] L. Liang , H. Shen , P. De Camilli , and J. S. Duncan , “A Novel Multiple Hypothesis Based Particle Tracking Method for Clathrin Mediated Endocytosis Analysis Using Fluorescence Microscopy,” IEEE Transactions on Image Processing 23, no. 4 (2014): 1844–1857, 10.1109/TIP.2014.2303633. PMC4373089 24808351

[28] C. Zhang , R. Liu , Z. Ding , et al., “Review of Machine Learning for Single‐Particle Tracking: Methods, Challenges, and Biophysical Insights,” Chemical & Biomedical Imaging 4, no. 5 (2026): 695–718, 10.1021/cbmi.5c00146. PMC13217339 42222567

[29] Y. Zhang and G. Yang , “A Motion Transformer for Single Particle Tracking in Fluorescence Microscopy Images,” in Medical Image Computing and Computer Assisted Intervention – MICCAI 2023, eds., H. Greenspan , A. Madabhushi , and P. Mousavi , et al., (Springer Nature Switzerland, 2023), 503–513, 10.1007/978-3-031-43993-3_49.

[30] Y. Zhang , D. Liu , and G. Yang , “Universal Transformer‐Based Tracker for Accurate Tracking of Particles and Cells in Microscopy,” preprint, bioRxiv, May 14, 2025, 10.1101/2025.05.09.653028.

[31] J. Pineda , B. Midtvedt , H. Bachimanchi , et al., “Geometric Deep Learning Reveals the Spatiotemporal Features of Microscopic Motion,” Nature Machine Intelligence 5, no. 1 (2023): 71–82, 10.1038/s42256-022-00595-0.

[32] C. Bi , K. L. Scrudders , Y. Zheng , M. Mahmoodi , S. T. Low‐Nam , and F. Huang , “BPS2025 ‐ SPTNet: A Deep‐Learning‐Based Framework for End‐to‐End Single‐Particle Tracking and Motion Dynamics Analysis,” preprint, bioRxiv, Feburary 8, 2025, 10.1101/2025.02.04.636521.

[33] M. Granfors , J. Pineda , B. Z. Gerbolés , J. B. Pereira , C. Manzo , and G. Volpe , “Global Graph Features Unveiled by Unsupervised Geometric Deep Learning,” Machine Learning: Science and Technology 7, no. 4 (2026): 045054, 10.48550/arXiv.2503.05560.

[34] H. Verdier , M. Duval , F. Laurent , A. Cassé , C. L. Vestergaard , and J.‐B. Masson , “Learning Physical Properties of Anomalous Random Walks Using Graph Neural Networks,” Journal of Physics A: Mathematical and Theoretical 54, no. 23 (2021): 234001, 10.1088/1751-8121/abfa45.

[35] J. Pineda , S. Masó‐Orriols , M. Masoliver , et al., “Enhanced Spatial Clustering of Single‐Molecule Localizations With Graph Neural Networks,” Nature Communications 16, no. 1 (2025): 9693, 10.1038/s41467-025-65557-7. PMC12583556 41184315

[36] A. Gentili and G. Volpe , “Characterization of Anomalous Diffusion Classical Statistics Powered by Deep Learning (CONDOR),” Journal of Physics A: Mathematical and Theoretical 54, no. 31 (2021): 314003, 10.1088/1751-8121/ac0c5d.

[37] H. D. Pinholt , S. S.‐R. Bohr , J. F. Iversen , W. Boomsma , and N. S. Hatzakis , “Single‐Particle Diffusional Fingerprinting: A Machine‐Learning Framework for Quantitative Analysis of Heterogeneous Diffusion,” Proceedings of the National Academy of Sciences 118, no. 31 (2021): 2104624118, 10.1073/pnas.2104624118. PMC8346862 34321355

[38] X. Feng , H. Sha , Y. Zhang , et al., “Reliable Deep Learning in Anomalous Diffusion Against out‐of‐Distribution Dynamics,” Nature Computational Science 4, no. 10 (2024): 761–772, 10.1038/s43588-024-00703-7. 39394501

[39] H. Seckler and R. Metzler , “Bayesian Deep Learning for Error Estimation in the Analysis of Anomalous Diffusion,” Nature Communications 13, no. 1 (2022): 6717, 10.1038/s41467-022-34305-6. PMC9640593 36344559

[40] E. Perez , F. Strub , H. De Vries , V. Dumoulin , and A. Courville , “FiLM: Visual Reasoning With a General Conditioning Layer,” Proceedings of the AAAI Conference on Artificial Intelligence 32, no. 1 (2018): 3942–3951, 10.1609/AAAI.V32I1.11671.

[41] A. Vaswani , N. Shazeer , N. Parmar , et al., “Attention Is All You Need,” Advances in Neural Information Processing Systems 30 (2017): 5998–6008.

[42] Y. Gal and Z. Ghahramani , “Dropout as a Bayesian Approximation: Representing Model Uncertainty in Deep Learning,” in Proceedings of the 33rd International Conference on Machine Learning (PMLR, 2016), 1050–1059.

[43] B. W. Silverman , Density Estimation for Statistics and Data Analysis (Routledge, 2018), 10.1201/9781315140919.

[44] G. Muñoz‐Gil , G. Volpe , M. A. Garcia‐March , et al., “Objective Comparison of Methods to Decode Anomalous Diffusion,” Nature Communications 12, no. 1 (2021): 6253, 10.1038/s41467-021-26320-w. PMC8556353 34716305

[45] X. Feng , Y. Jiang , H. Sha , et al., “Enhancing Diffusion Estimation in Single‐Particle Experiments Through Motion Change Analysis Using Deep Learning,” Journal of Physics: Photonics 7, no. 3 (2025): 035006, 10.1088/2515-7647/add978.

[46] V. Ulman , M. Maska , K. E. G. Magnusson , et al., “An Objective Comparison of Cell‐Tracking Algorithms,” Nature Methods 14, no. 12 (2017): 1141–1152, 10.1038/nmeth.4473. PMC5777536 29083403

[47] B. Midtvedt , S. Helgadottir , A. Argun , J. Pineda , D. Midtvedt , and G. Volpe , “Quantitative Digital Microscopy With Deep Learning,” Applied Physics Reviews 8, no. 1 (2021): 011310, 10.1063/5.0034891.

[48] C. Schirripa Spagnolo , A. Moscardini , R. Amodeo , F. Beltram , and S. Luin , “Optimized Two‐Color Single‐Molecule Tracking of Fast‐Diffusing Membrane Receptors,” Advanced Optical Materials 12, no. 9 (2024): 2302012, 10.1002/adom.202302012.

[49] A. Ghosal , Y.‐H. Wang , N. Nguyen , L. Troyer , and S. Kim , “Practical Considerations for Accurate Estimation of Diffusion Parameters From Single‐Particle Tracking in Living Cells,” The Journal of Chemical Physics 163, no. 13 (2025): 134202, 10.1063/5.0284172. PMC12670385 41051298

[50] C. S. Spagnolo and S. Luin , “Impact of Temporal Resolution in Single Particle Tracking Analysis,” Discover Nano 19, no. 1 (2024): 87, 10.1186/s11671-024-04029-1. PMC11082114 38724858

[51] A. Driouchi , M. Bretan , B. J. Davis , et al., “Oblique Line Scan Illumination Enables Expansive, Accurate and Sensitive Single‐Protein Measurements in Solution and in Living Cells,” Nature Methods 22, no. 3 (2025): 559–568, 10.1038/s41592-025-02594-6. PMC11903300 39966678

[52] N.‐S. Cheng , “Formula for the Viscosity of a Glycerol−Water Mixture,” Industrial & Engineering Chemistry Research 47, no. 9 (2008): 3285–3288, 10.1021/ie071349z.

[53] H. C. Berg , “Random Walks in Biology,” (Princeton Science Library, Princeton University Press, Princeton, NJ, 2025).

[54] A. Einstein , “Über Die von der Molekularkinetischen Theorie der Wärme Geforderte Bewegung von in Ruhenden Flüssigkeiten Suspendierten Teilchen,” Annalen der Physik 322, no. 8 (1905): 549–560, 10.1002/andp.19053220806.

[55] F. Höfling and T. Franosch , “Anomalous Transport in the Crowded World of Biological Cells,” Reports on Progress in Physics 76, no. 4 (2013): 046602, 10.1088/0034-4885/76/4/046602. 23481518

[56] A. Sabri , X. Xu , D. Krapf , and M. Weiss , “Elucidating the Origin of Heterogeneous Anomalous Diffusion in the Cytoplasm of Mammalian Cells,” Physical Review Letters 125, no. 5 (2020): 058101, 10.1103/PhysRevLett.125.058101. 32794890

[57] J.‐H. Jeon , A. V. Chechkin , and R. Metzler , “Scaled Brownian Motion: A Paradoxical Process With a Time Dependent Diffusivity for the Description of Anomalous Diffusion,” Physical Chemistry Chemical Physics 16, no. 30 (2014): 15811–15817, 10.1039/C4CP02019G. 24968336

[58] R. Metzler , J.‐H. Jeon , A. G. Cherstvy , and E. Barkai , “Anomalous Diffusion Models and Their Properties: Non‐Stationarity, Non‐Ergodicity, and Ageing at the Centenary of Single Particle Tracking,” Physical Chemistry Chemical Physics 16, no. 44 (2014): 24128–24164, 10.1039/C4CP03465A. 25297814

[59] H. Zhang , M. Cisse , Y. N. Dauphin , and D. Lopez‐Paz , “Mixup: Beyond Empirical Risk Minimization,” in International Conference on Learning Representations (2018).

[60] A. Pacheco‐Pozo , M. Balcerek , A. Wyłomańska , K. Burnecki , I. M. Sokolov , and D. Krapf , “Langevin Equation in Heterogeneous Landscapes: How to Choose the Interpretation,” Physical Review Letters 133, no. 6 (2024): 067102, 10.1103/PhysRevLett.133.067102. 39178429

[61] Y. Zhang , J. Zhu , H. Xie , and Y. He , “Physics‐Informed Deep Learning for Stochastic Particle Dynamics Estimation,” Proceedings of the National Academy of Sciences 122, no. 9 (2025): 2418643122, 10.1073/pnas.2418643122. PMC11892597 40014572

[62] Y. Song , J. Sohl‐Dickstein , D. P. Kingma , A. Kumar , S. Ermon , and B. Poole , “Score‐Based Generative Modeling Through Stochastic Differential Equations,” in International Conference on Learning Representations (2021).

[63] G. Fernández‐Fernández , C. Manzo , M. Lewenstein , A. Dauphin , and G. Muñoz‐Gil , “Learning Minimal Representations of Stochastic Processes With Variational Autoencoders,” Physical Review E 110 (2024): L012102, 10.1103/PhysRevE.110.L012102. 39160967

[64] I. Sgouralis , L. W. Q. Xu , A. P. Jalihal , Z. Kilic , N. G. Walter , and S. Pressé , “BNP‐Track: A Framework for Superresolved Tracking,” Nature Methods 21, no. 9 (2024): 1716–1724, 10.1038/s41592-024-02349-9. PMC11399105 39039336

[65] Y. Jiang , H. Sha , S. Liu , P. Qin , and Y. Zhang , “AutoUnmix: An Autoencoder‐Based Spectral Unmixing Method for Multi‐Color Fluorescence Microscopy Imaging,” Biomedical Optics Express 14, no. 9 (2023): 4814–4827, 10.1364/BOE.498421. PMC10545201 37791286

[66] H. Sha , H. Li , Y. Zhang , and S. Hou , “Deep Learning‐Enhanced Single‐Molecule Spectrum Imaging,” APL Photonics 8, no. 9 (2023): 096102, 10.1063/5.0156793.

[67] P. Veličković , G. Cucurull , A. Casanova , A. Romero , P. Liò , and Y. Bengio , “Graph Attention Networks,” in International Conference on Learning Representations (2018).

[68] K. Bernardin and R. Stiefelhagen , “Evaluating Multiple Object Tracking Performance: The CLEAR MOT Metrics,” EURASIP Journal on Image and Video Processing 2008 (2008): 246309, 10.1155/2008/246309.

[69] M. Balcerek , A. Wyłomańska , K. Burnecki , R. Metzler , and D. Krapf , “Modelling Intermittent Anomalous Diffusion With Switching Fractional Brownian Motion,” New Journal of Physics 25, no. 10 (2023): 103031, 10.1088/1367-2630/ad00d7.

[70] B. B. Mandelbrot and J. W. Van Ness , “Fractional Brownian Motions, Fractional Noises and Applications,” SIAM Review 10, no. 4 (1968): 422–437, 10.1137/1010093.

