# Supplementary material for: ReLink: Reliable Deep Learning for Microscopic Motion Tracking
Source: Adv Sci (Weinh). 2026 Oct 28:e78541. Online ahead of print. doi: 10.1002/advs.78541 (PMC13717127; doi:10.1002/advs.78541)
Supplement: Supplementary file 1 [file ADVS-9999-e78541-s001.pdf]

Supplementary Information

**Section**	**Page**
**Figures**	
Supplementary Figure 1: Trajectory-linking performance under different frame lengths	1
Supplementary Figure 2: Trajectory-linking performance under different particle densities	2
Supplementary Figure 3: Controlled decomposition of localization and detection errors in trajectory linking	3
Supplementary Figure 4: Trajectory-linking performance under different signal-to-noise ratios	4
Supplementary Figure 5: Trajectories obtained from the red-boxed region in Fig. 3a after confidence-based filtering.	5
Supplementary Figure 6: Confidence-based filtering under different motion models	6
Supplementary Figure 7: Performance comparison in AnDi 1 Challenge	7
Supplementary Figure 8: Diffusion of 200 nm beads in glycerol–water mixtures	8
Supplementary Figure 9: Diffusion of 30 nm beads in glycerol–water mixtures	9
Supplementary Figure 10: Anomalous exponent estimation in glycerol–water mixtures	10
Supplementary Figure 11: Confusion matrices for three-stage classification during hydrogel gelation	11
Supplementary Figure 12: Ablation study of anomalous diffusion analysis for quantum dots in L929 cells	12
Supplementary Figure 13: Effectiveness of the physical statistics module during training	13
Supplementary Figure 14: Representation of network architecture	14
Supplementary Figure 15: Schematic representation of microscope for single-particle tracking	15
**Tables**	
Supplementary Table 1: Dunnett-adjusted comparisons of ReLink with competing linking methods across particle densities	16
Supplementary Table 2: Dunnett-adjusted comparisons of ReLink with competing linking methods in noisy-video simulations	17
Supplementary Table 3: Effects of adaptive and fixed confidence thresholds relative to no confidence filtering	18
Supplementary Table 4: Effect of the physical statistics module across confidence-filtering settings on MOTA	19

Supplementary Table 5: Effect of adaptive confidence filtering in challenging video-based simulations	20
Supplementary Table 6: Effect of adaptive confidence filtering under fast-diffusion conditions	21
Supplementary Table 7: Comparison of evaluation metrics under different confidence thresholds for AnDi 2 Benchmark	22
Supplementary Table 8: Post-processing parameters for trajectory reconstruction	23
Supplementary Table 9: Network architecture and training hyperparameters fixed across all datasets	24
**Algorithm**	
Supplementary Algorithm 1: Adaptive confidence filtering with Monte Carlo dropout and KDE valley threshold	25
**Notes**	
Supplementary Note 1: Split event detection strategy in post-processing stage	26
Supplementary Note 2: Detailed parameter settings for generating simulated datasets.	27
Supplementary Note 3: Detailed settings for public benchmark datasets	28
Supplementary Note 4: Home-built microscope for single-particle tracking	29
Supplementary Note 5: Experimental protocols	30

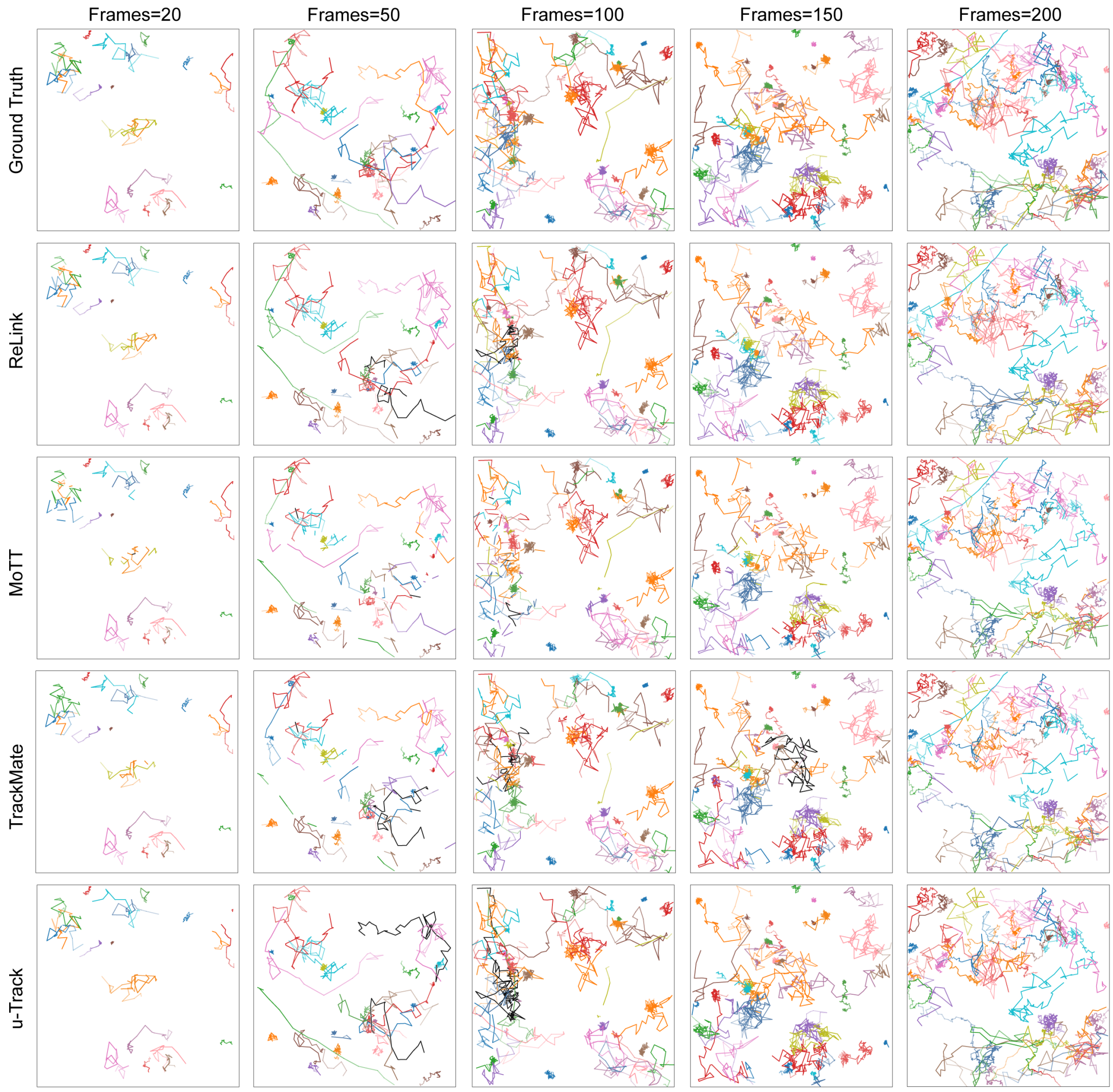

Supplementary Figure 1. Trajectory-linking performance under different frame lengths. Comparison of trajectory-linking accuracy on simulated data under input sequence lengths of 20, 50, 100, 150 and 200 frames with particle density of 20 particles/frame within a fixed 64 × 64-pixel FOV. Trajectories reconstructed by ReLink, MoTT[1], TrackMate[2] and u-Track[3] are compared with ground truth.

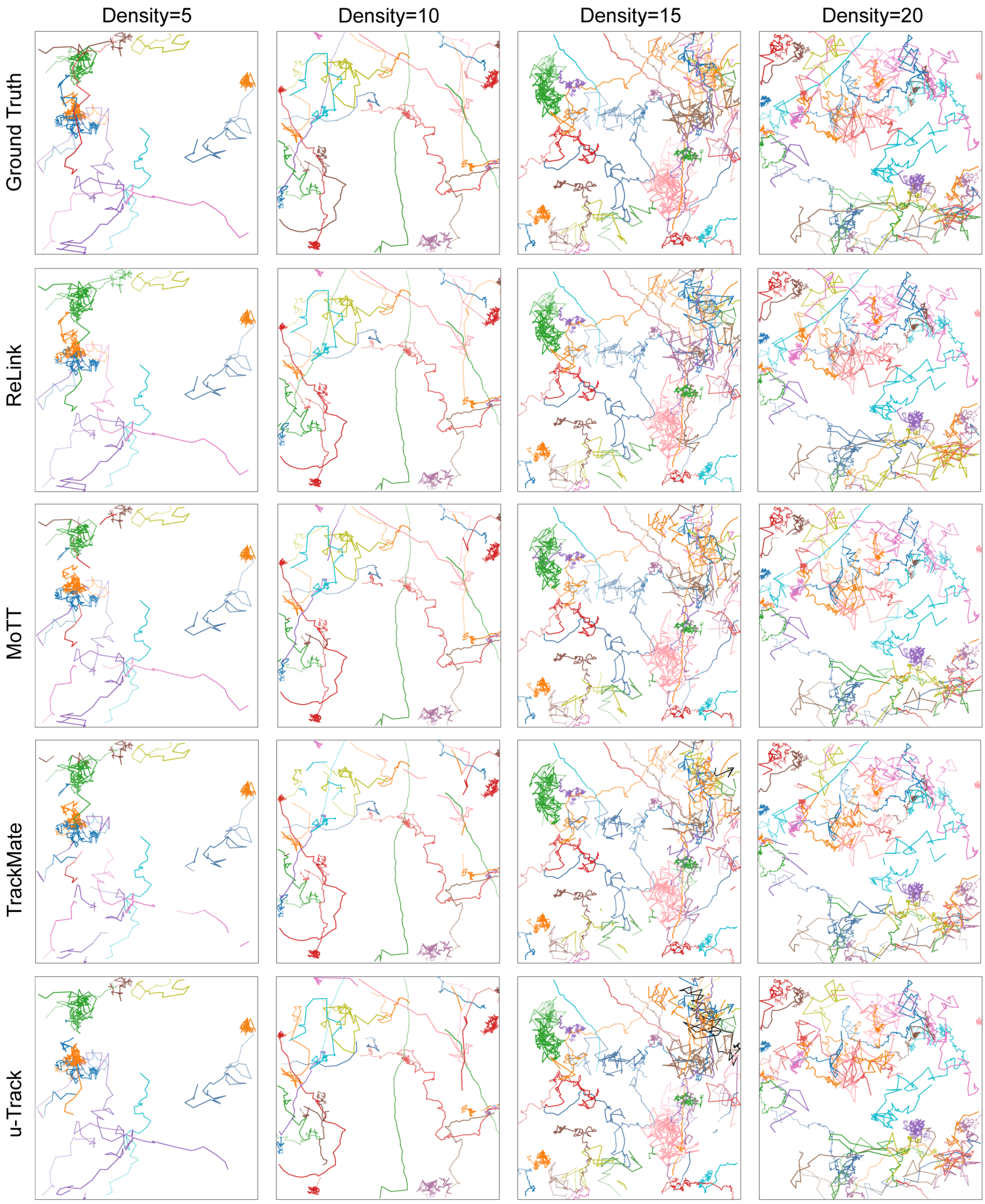

Supplementary Figure 2. Trajectory-linking performance under different particle densities. Comparison of trajectory-linking accuracy on simulated data with varying particle densities (5, 10, 15 and 20 particles/frame within a 64 × 64 FOV) and 200 frames. Trajectories reconstructed by ReLink, MoTT, TrackMate and u-Track are compared with ground truth.

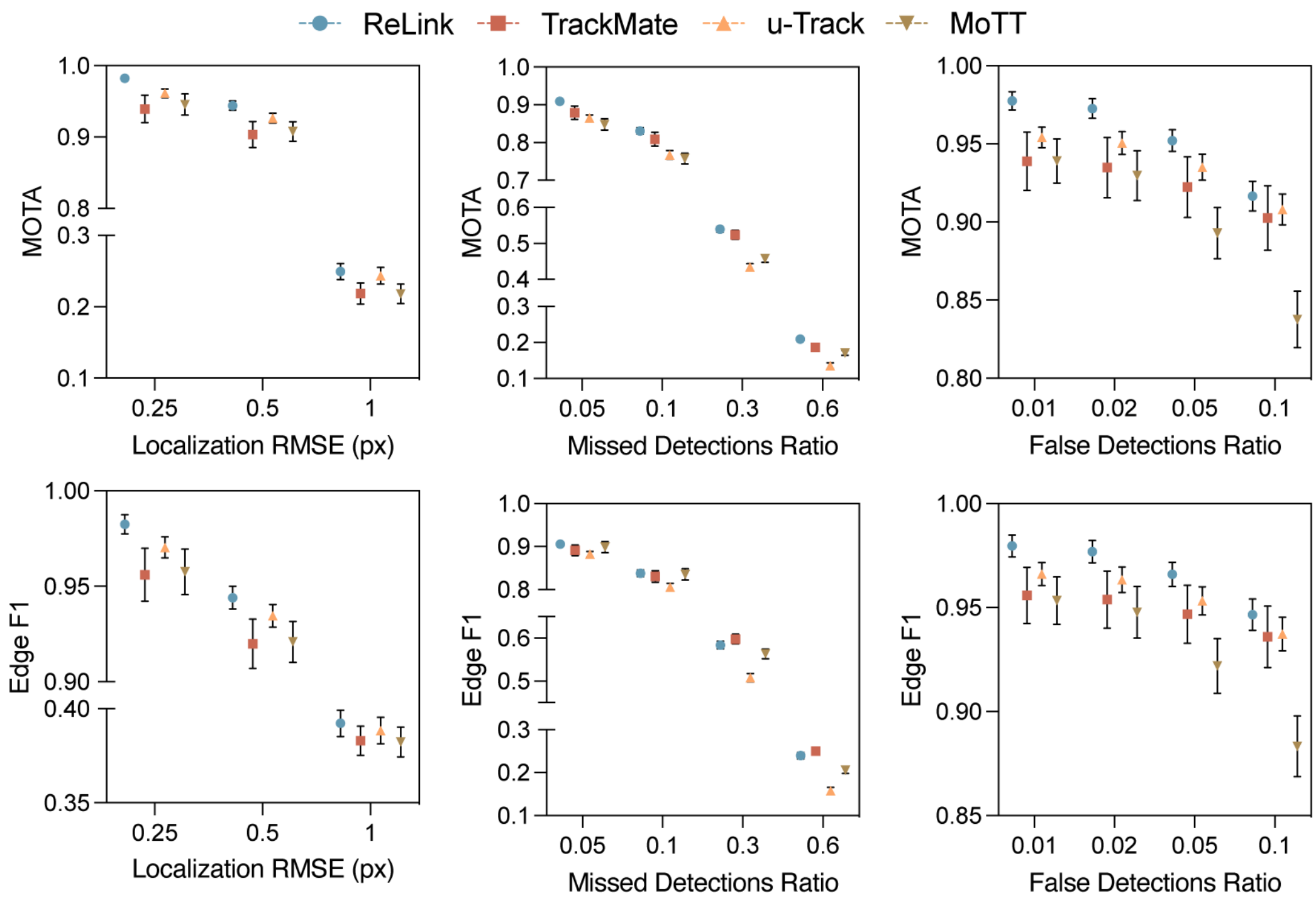

Supplementary Figure 3. Controlled decomposition of localization and detection errors in trajectory linking. MOTA (top row) and Edge F1 score (bottom row) of ReLink, MoTT, TrackMate and u-Track under independently controlled localization errors (left), missed detections (middle) and false detections (right). Localization errors are introduced by perturbing the particle coordinates to obtain localization root mean square errors (RMSEs) of 0.25, 0.5 and 1 pixel. Missed detections are generated by randomly removing 5%, 10%, 30% and 60% of the ground-truth localizations, whereas false detections are generated by adding spurious localization points at ratios of 1%, 2%, 5% and 10% relative to the ground-truth localizations. Only the indicated error source is varied in each experiment, while the underlying ground-truth trajectories and all other simulation conditions are held fixed. All methods are evaluated using the same perturbed localization inputs. Each simulated sequence contains 10 particles/frame within 64 × 64-pixel FOV (2.44×10^{-3} particles/px^2) and spans 50 frames. For consistency with the other evaluations, a 1-pixel spatial tolerance is used for detection-to-detection matching for all methods; therefore, when the localization RMSE approaches 1 pixel, part of the decrease in the reported metrics also reflects perturbed detections falling outside this matching tolerance. Points represent the mean over n = 55 independent simulated sequences, and error bars indicate 95% confidence intervals. Broken y-axes are used in the localization-error and missed-detection panels to resolve both the high- and low-performance ranges.

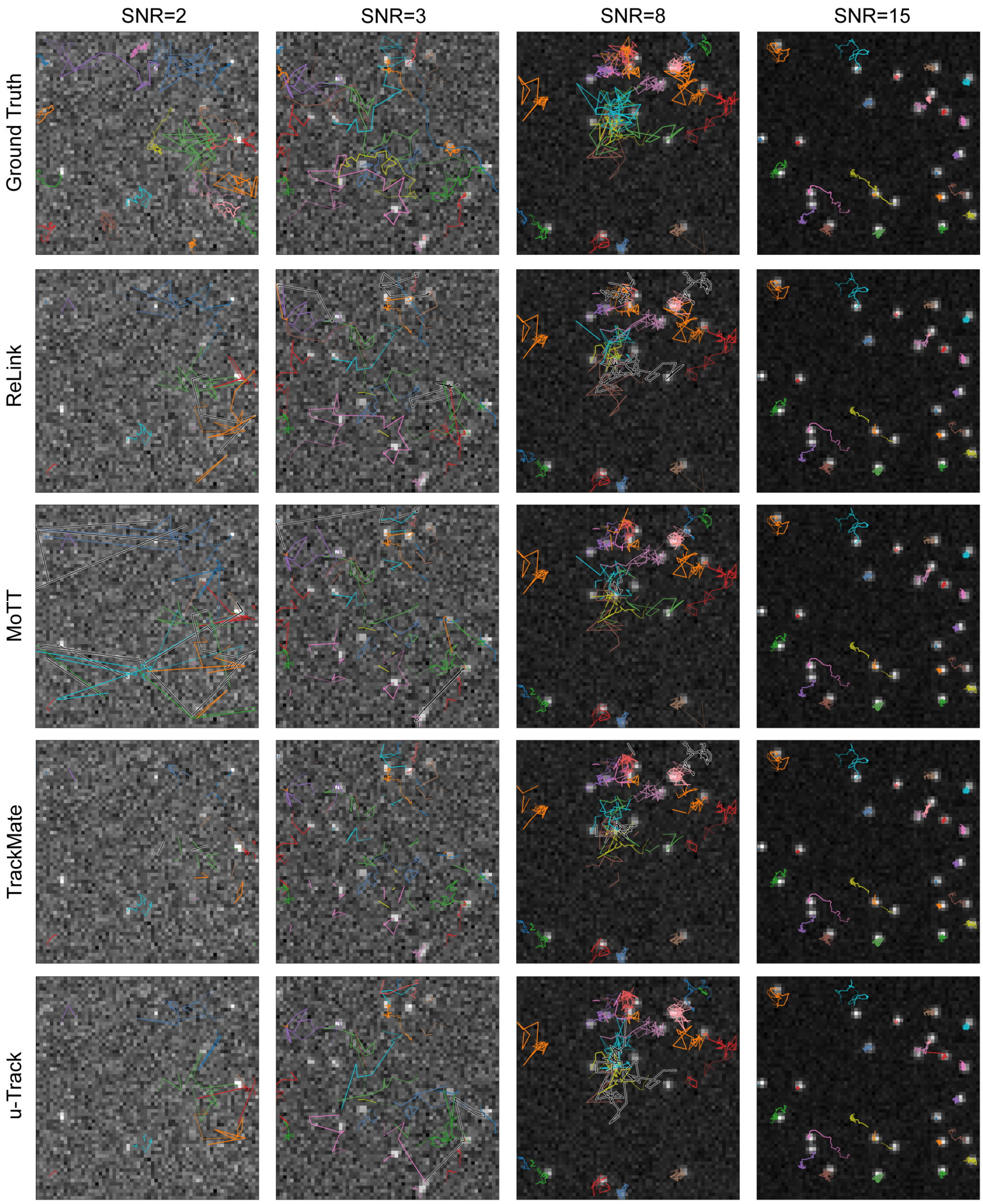

Supplementary Figure 4. Trajectory-linking performance under different signal-to-noise ratios. Comparison of reconstructed trajectories under different signal-to-noise ratios (SNR = 2, 3, 8, 15) with 50 frames and 10 particles/frame within a fixed 64 × 64-pixel FOV. Trajectories obtained by ReLink, MoTT, TrackMate and u-Track are compared with ground truth.

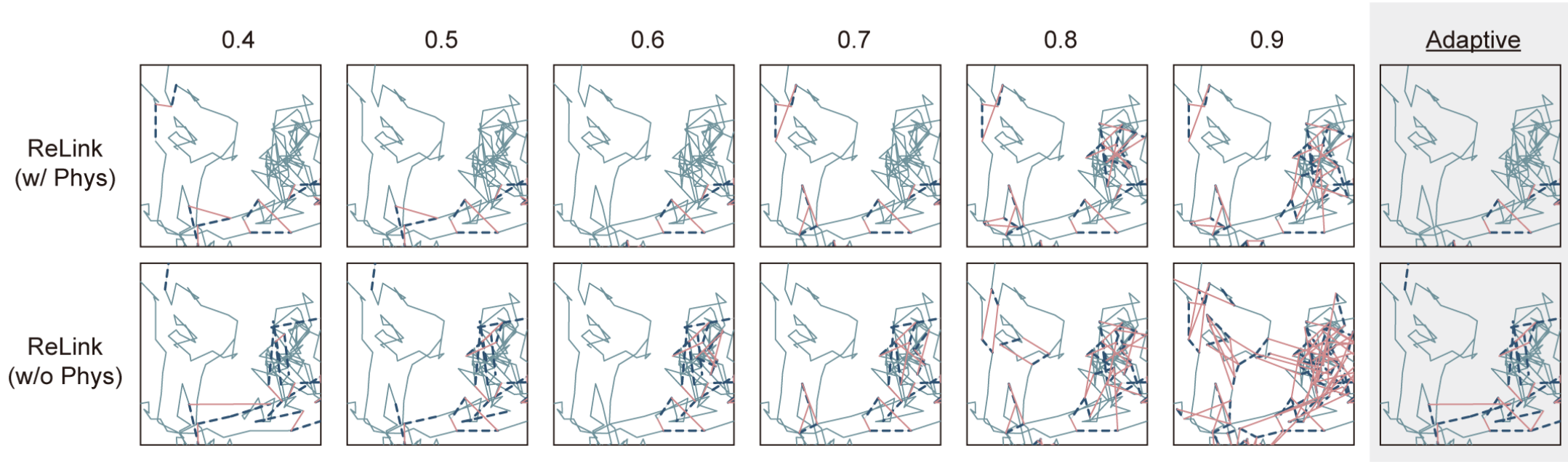

Supplementary Figure 5. Trajectories obtained from the red-boxed region in Fig. 3a after confidence-based filtering. Correctly predicted links are shown in blue-green, incorrect links in red, and missed ground-truth links as dark-blue dashed lines. Results obtained by ReLink with and without the physical statistics module (Phys) under different confidence thresholds (0.4, 0.5, 0.6, 0.7, 0.8, 0.9 and the adaptive threshold) are shown for comparison.

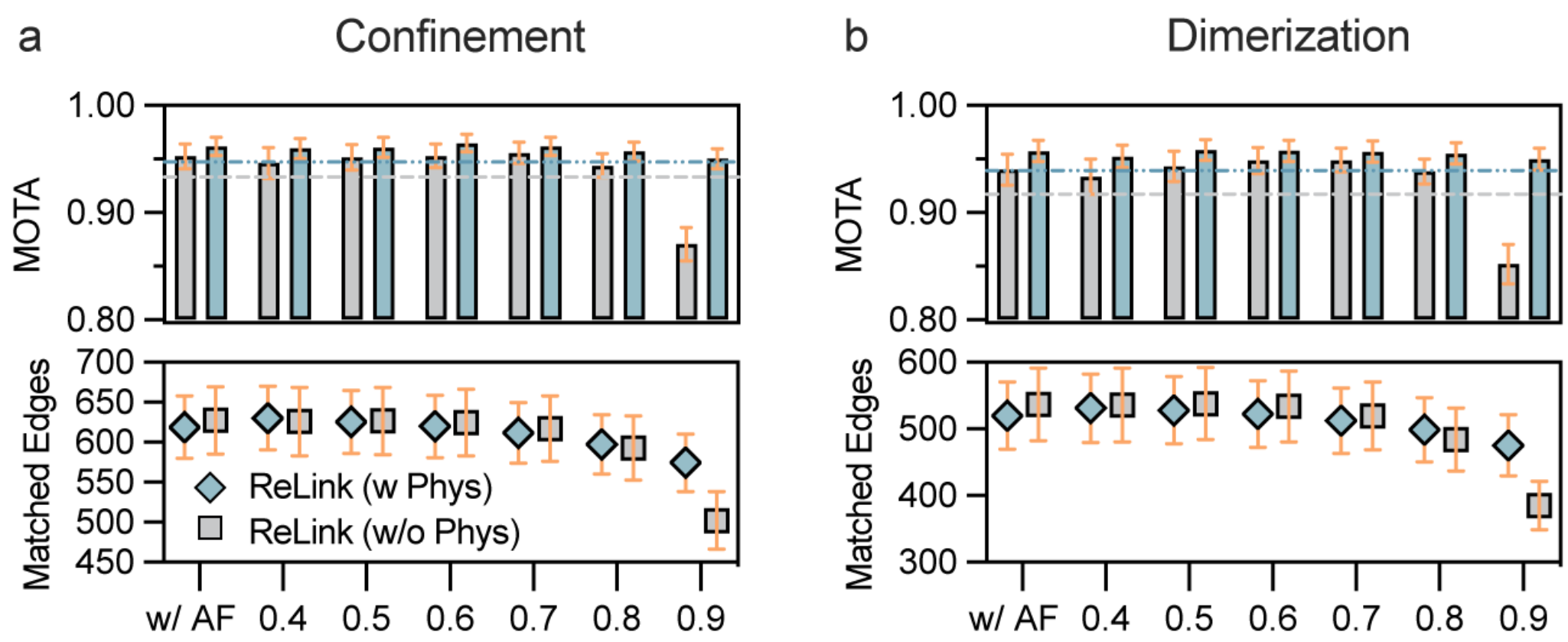

Supplementary Figure 6. Confidence-based filtering under different motion models. a, b, Evaluations on confinement (**a**) and dimerization (**b**) motion models. MOTA and the number of correctly matched edges are shown across fixed confidence thresholds and the adaptive threshold, with and without the physical statistics module (Phys). Here, "w/ AF" denotes ReLink with the adaptive filtering module using its default adaptive threshold, while numerical labels indicate fixed confidence thresholds. Dashed lines represent the corresponding results without confidence-based filtering (w/o AF). Each video in the dataset involves 15 particles/frame within a fixed 64 × 64-pixel FOV, 50 frames and diffusion coefficients ranging from 10^0 to 10^1 px^2/frame. Data are presented as mean with 95% confidence interval (n = 55 independently simulated sequences per setting).

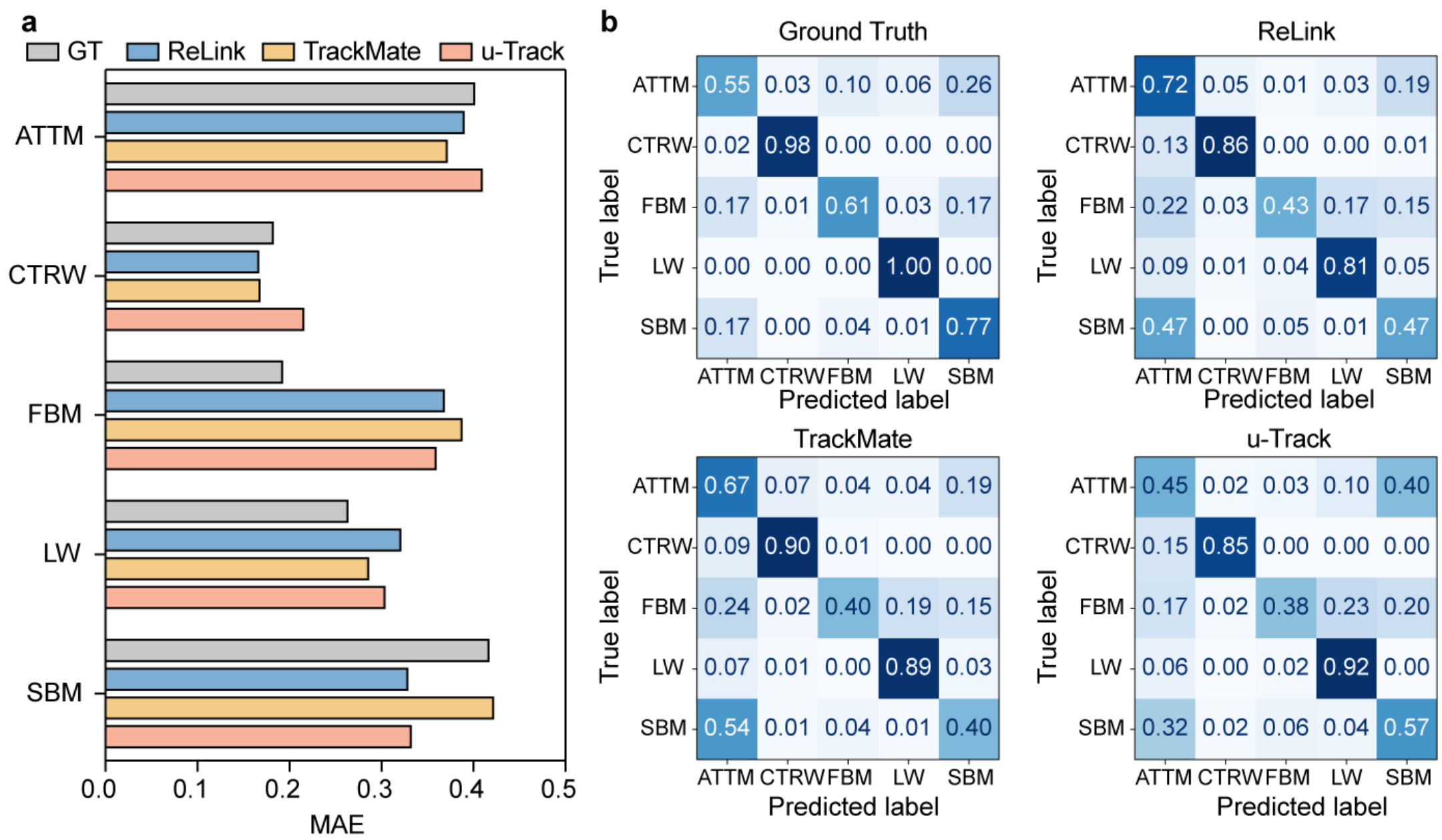

Supplementary Figure 7. Performance comparison in AnDi 1 Challenge. a, Mean absolute error (MAE) of anomalous exponent (α) prediction across different motion models, including annealed transient time motion (ATTM), continuous-time random walk (CTRW), fractional Brownian motion (FBM), Lévy walk (LW) and scaled Brownian motion (SBM) models, using trajectories reconstructed by Ground Truth (GT), ReLink, TrackMate and u-Track. **b,** Confusion matrices for diffusion-model classification based on trajectories reconstructed by GT, ReLink, TrackMate and u-Track.

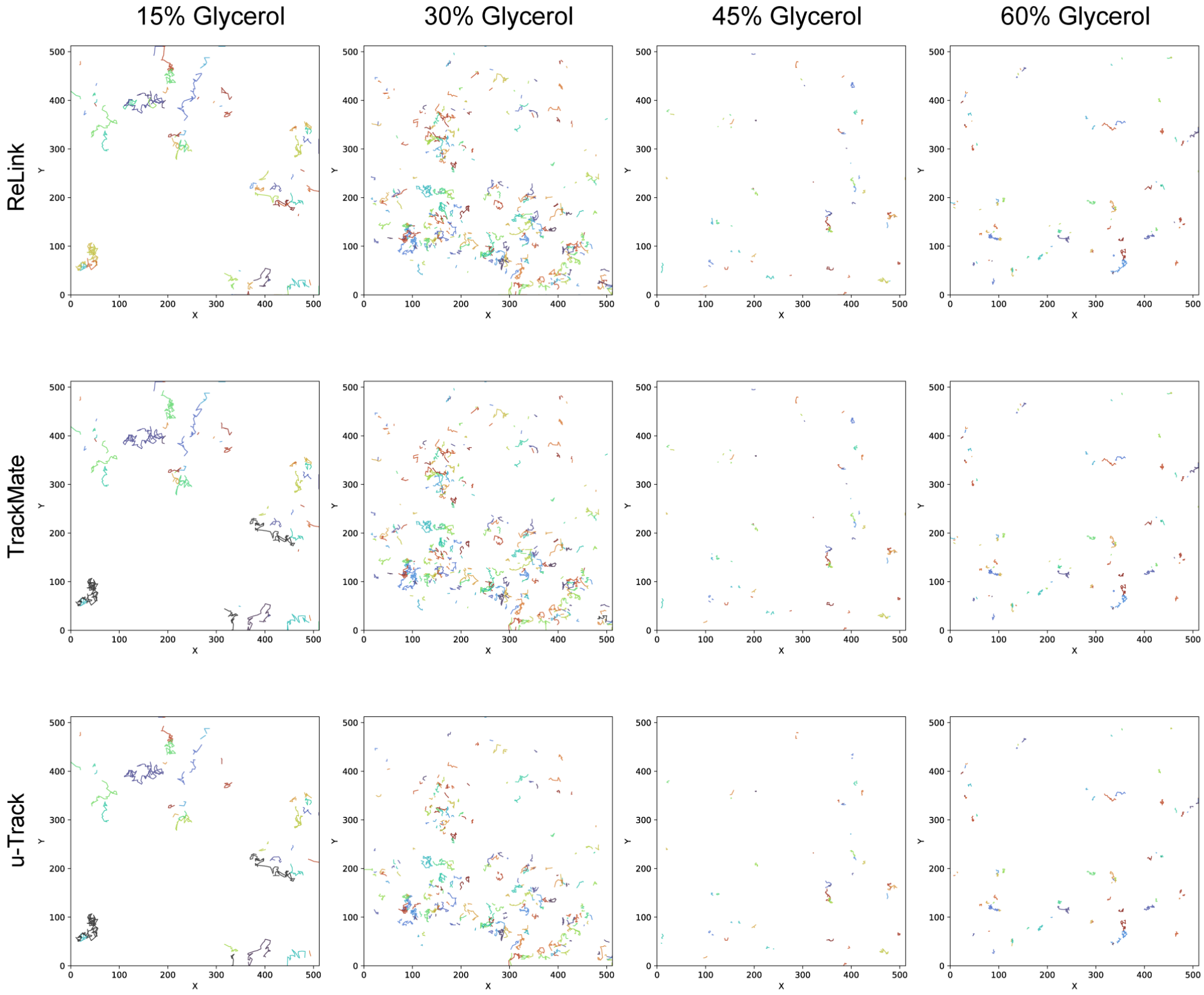

Supplementary Figure 8. Diffusion of 200 nm beads in glycerol–water mixtures. Diffusion trajectories of 200 nm fluorescent beads reconstructed by ReLink, TrackMate and u-Track at glycerol volume fractions of 15%, 30%, 45% and 60%. Videos for 15%, 30%, 45% and 60% contain 200 frames.

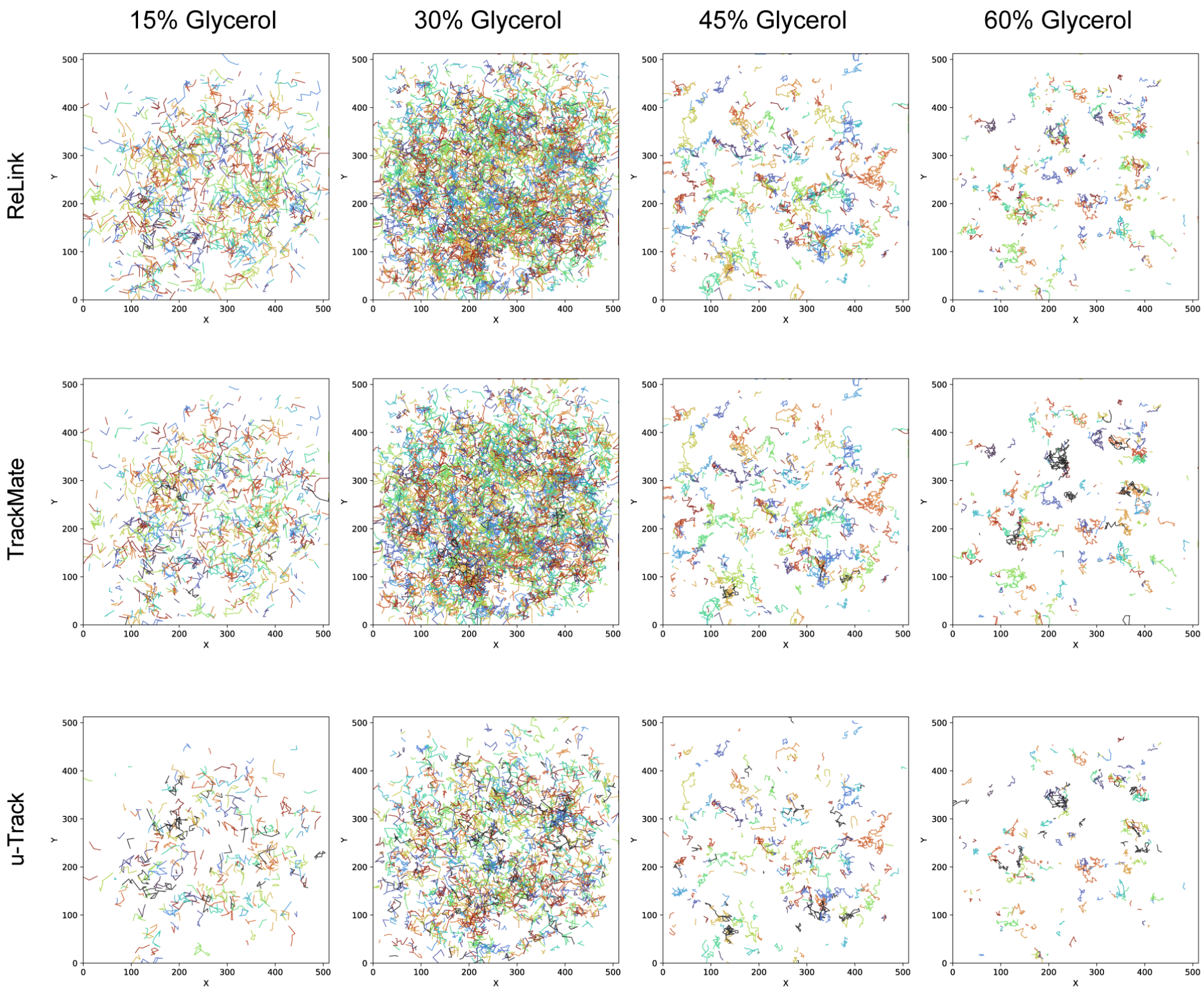

Supplementary Figure 9. Diffusion of 30 nm beads in glycerol–water mixtures. Diffusion trajectories of 30 nm fluorescent beads reconstructed by ReLink, TrackMate and u-Track at glycerol volume fractions of 15%, 30%, 45% and 60%. Videos for 15%, 45% and 60% contain 200 frames while videos for 30% contain 1000 frames.

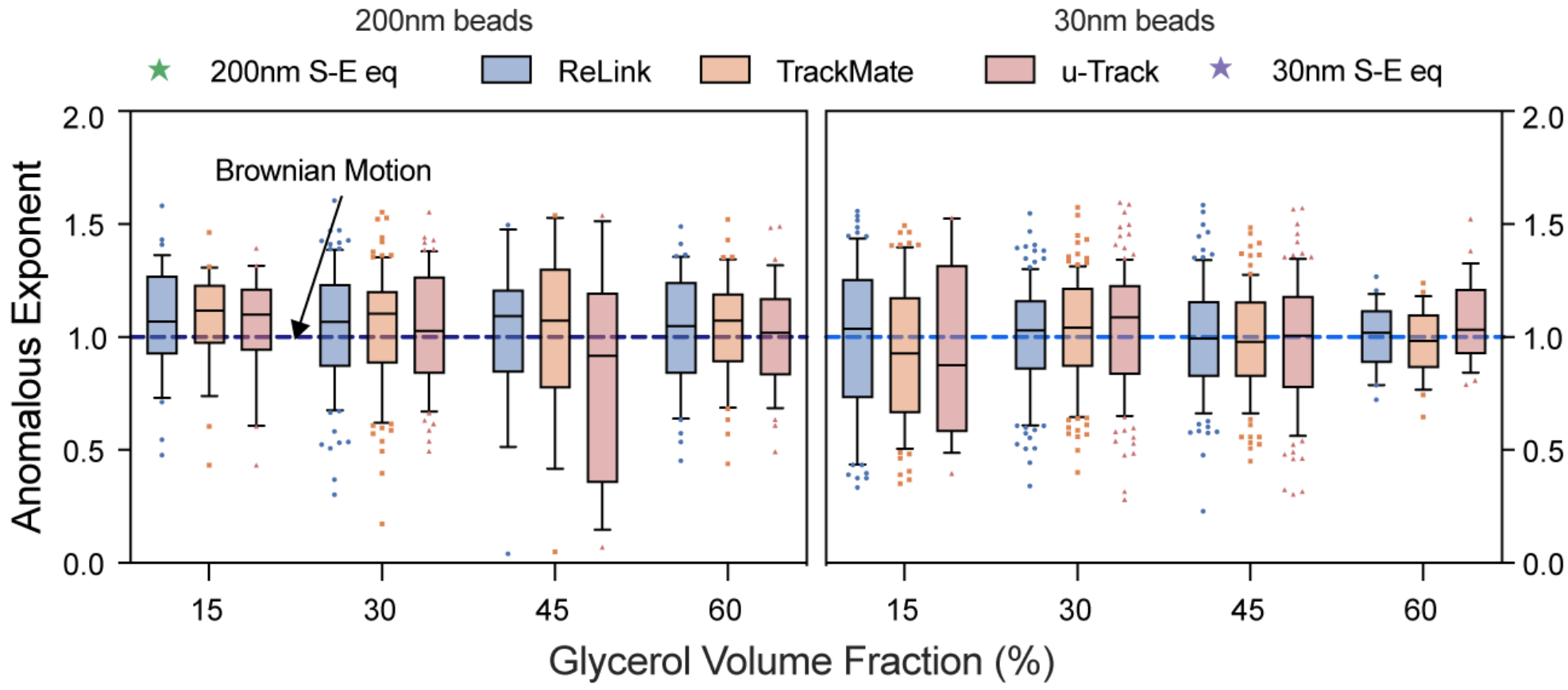

Supplementary Figure 10. Anomalous exponent estimation in glycerol–water mixtures. Anomalous exponent α predictions by U-LFormer[4] for 200 nm and 30 nm fluorescent beads in glycerol–water mixtures under 15%–60% glycerol volume fraction, based on trajectories reconstructed by ReLink, TrackMate and u-Track. All methods yield α values around 1, consistent with Brownian diffusion. Box plots show the median (center line), interquartile range (box), and the 10th–90th percentiles (whiskers). Points outside this range indicate the tails of the distribution rather than statistical outliers.

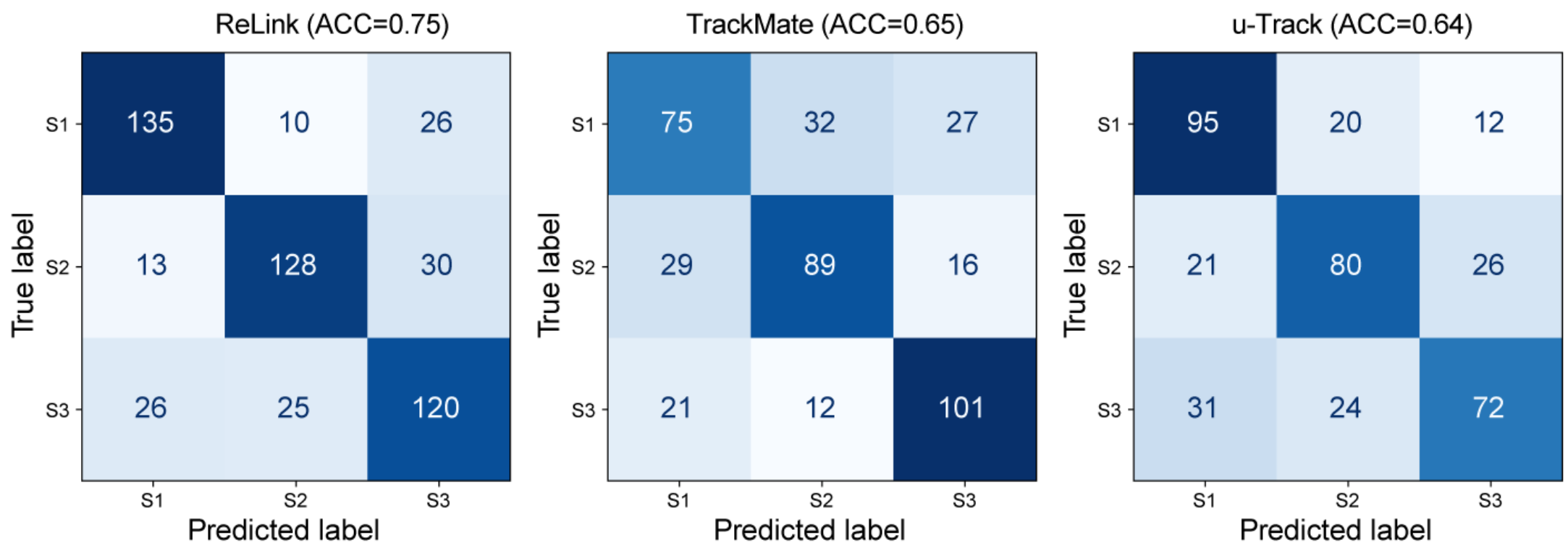

Supplementary Figure 11. Confusion matrices for three-stage classification during hydrogel gelation. Trajectories linked by ReLink show classification accuracy (0.75) compared with TrackMate (0.65) and u-Track (0.64). S1, S2, S3 denote gelation Stage 1, Stage 2 and Stage 3, respectively, corresponding to Fig. 5.

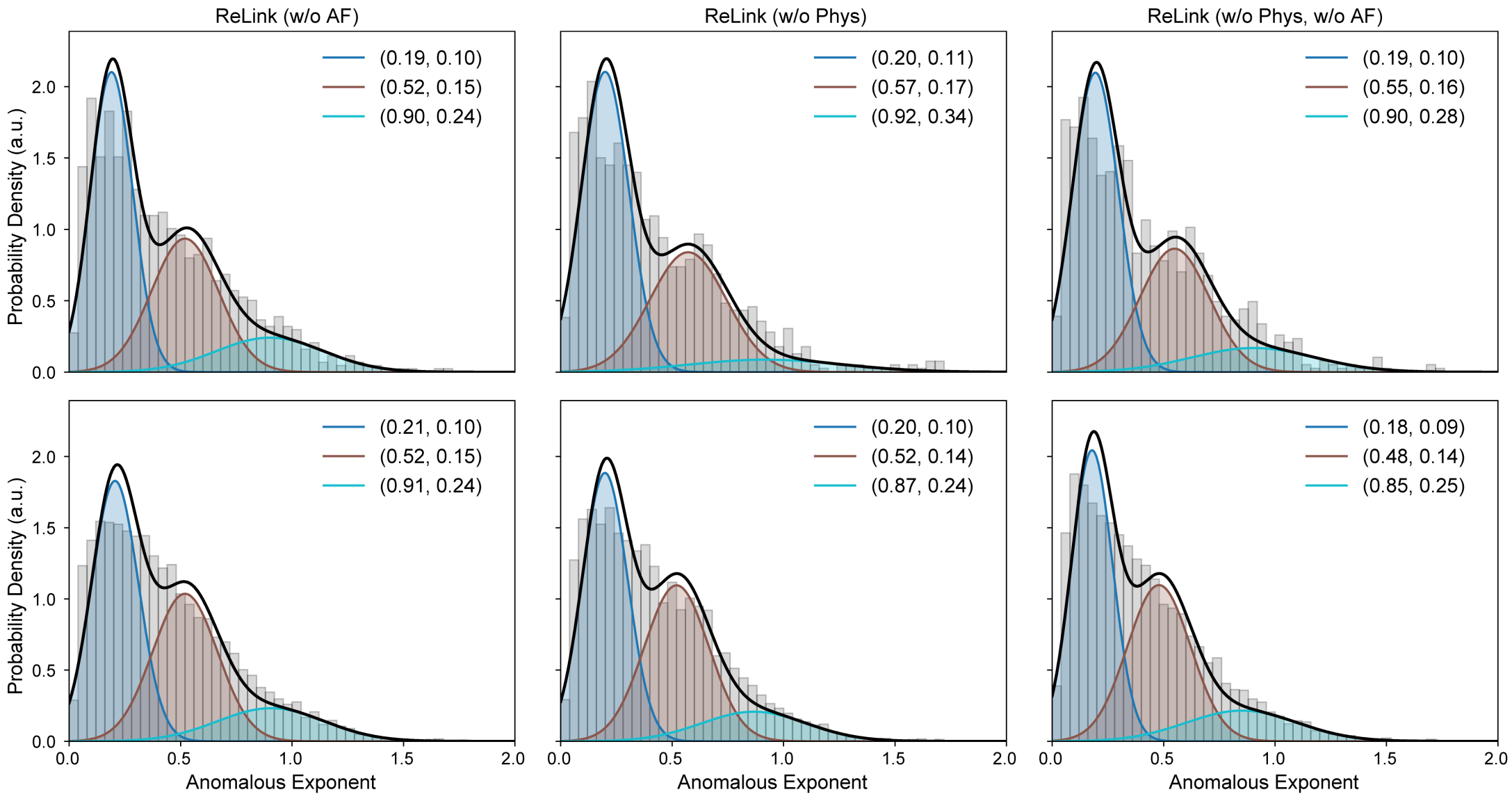

Supplementary Figure 12. Ablation study of anomalous diffusion analysis for quantum dots in L929 cells. Histograms of anomalous exponents (α) and fitted Gaussian mixture models (GMMs) for ReLink without adaptive filtering module (w/o AF), without the physical statistics module (w/o Phys), and without both modules (w/o Phys, w/o AF) under low-density (top) and high-density (bottom) conditions. Legends report the mean and standard deviation of each GMM component.

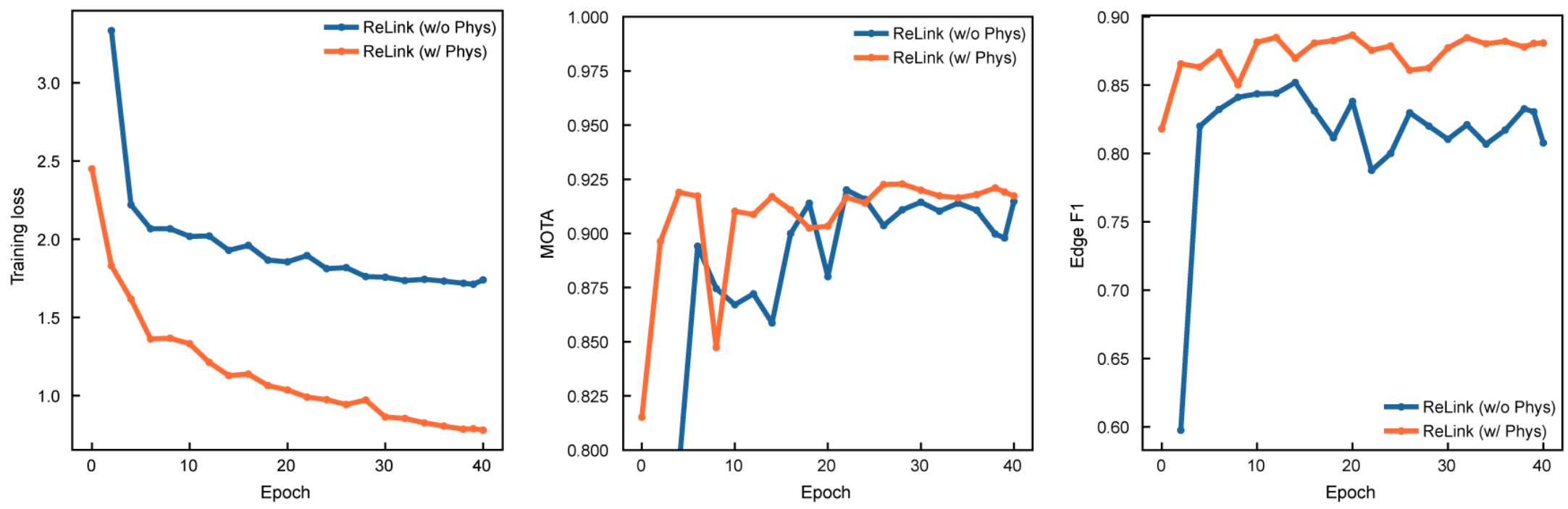

Supplementary Figure 13. Effectiveness of the physical statistics module during training. Training loss, MOTA and Edge F1 metrics on the training set for models with and without the physical statistics module.

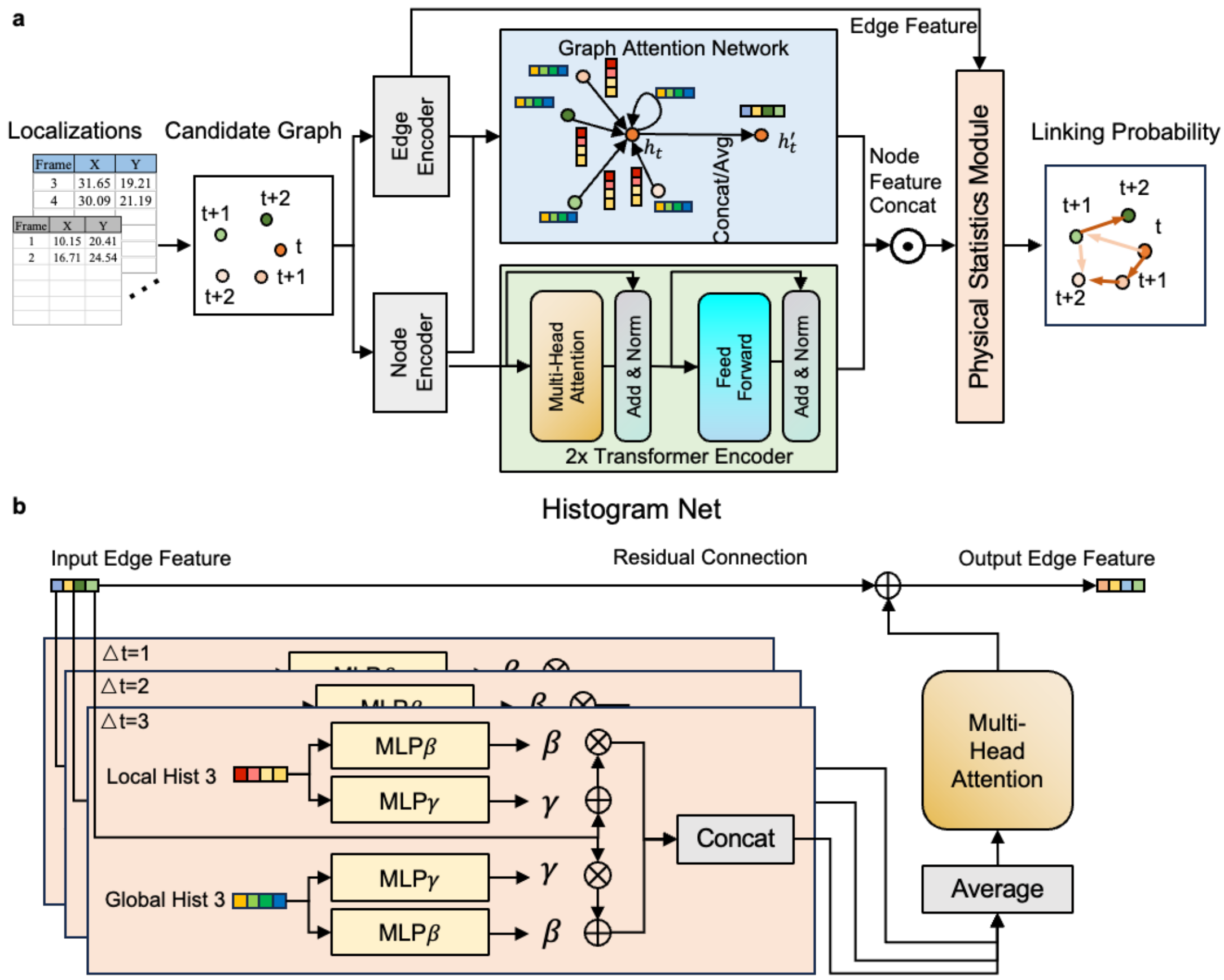

Supplementary Figure 14. Representation of network architecture. a. Physics-informed GNN architecture. Candidate graphs are first constructed from particle localizations across consecutive frames. Node and edge attributes are separately encoded using multi-layer perceptrons (MLPs) to obtain initial node and edge features. Local node representations are updated through graph attention layers (GATs)[5] that incorporate edge embeddings during message passing, while global contextual information is captured using a Transformer[6] encoder applied to node embeddings. The physical statistics module incorporates scores derived from local mean jump distances and global jump-distance distributions into node features through a Score Net, while integrating local and global jump-distance histograms into edge features through feature-wise linear modulation (FiLM) and multi-head attention across multiple temporal scales. Finally, an MLP decoder predicts the connection probability for each candidate edge. Detailed descriptions are provided in the Methods section. The architecture of physical statistics module is illustrated in Fig. 1c. **b.** Architecture of the Histogram Net in the physical statistics module. For each time lag Δt, local and global histograms are independently processed by multi-layer perceptrons (MLPs) to generate scale-specific modulation parameters γ and β, which are applied to the input edge features as feature-wise linear modulation (FiLM)[7]. The resulting multi-scale features are aggregated across multiple scales and subsequently fused using a multi-head attention mechanism to capture inter-scale dependencies. A residual connection is finally employed to produce the output edge features enriched with fused statistical information.

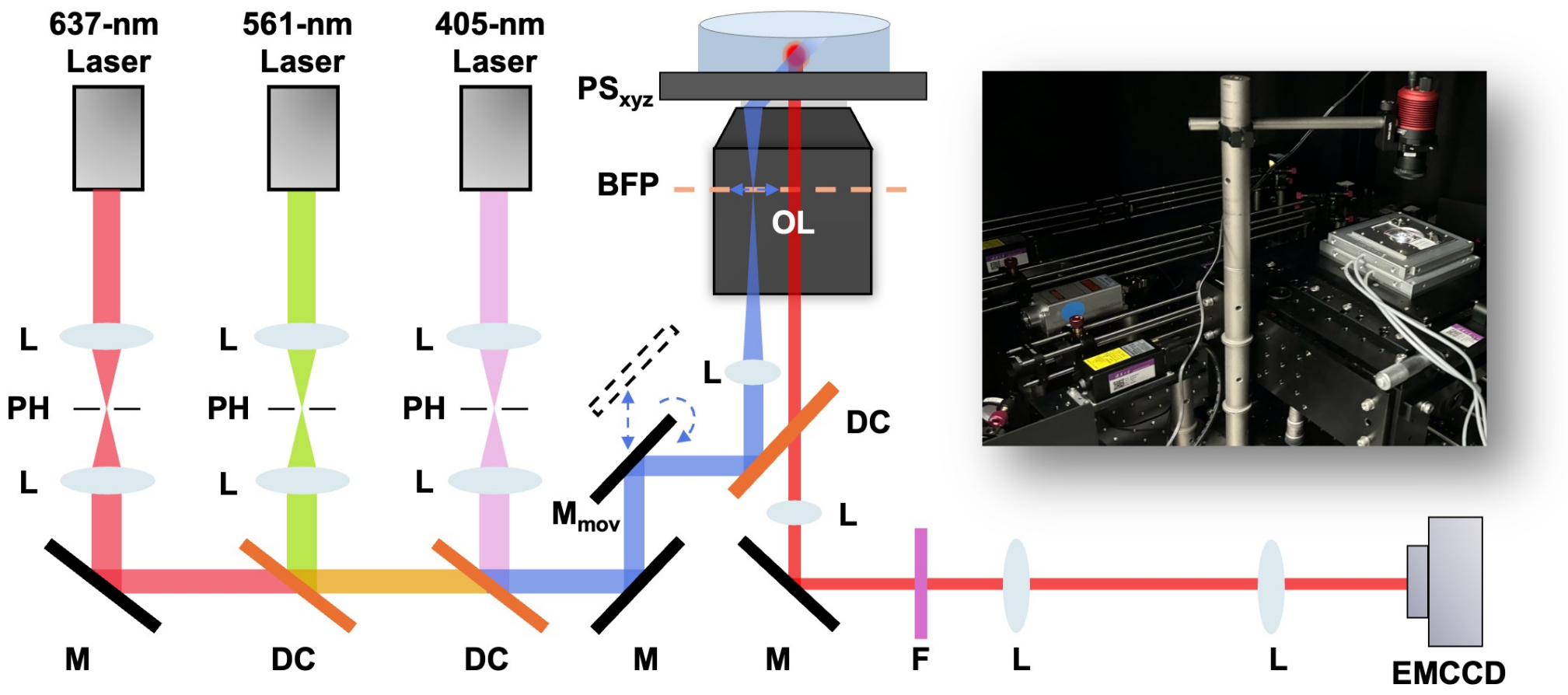

Supplementary Figure 15. Schematic representation of microscope for single-particle tracking. L: lens; M: mirror; M_{mov}: movable mirror with translation stage; PH: pinhole; DC: dichroic mirror; OL: objective lens; PS_{xyz}: XYZ Piezoelectric stage; BFP: back focal plane; F: fluorescence filter.

Metrics	Density (particles /frame)	Comparison	Mean Diff.	95% CI	Significant?	Adjusted *P* Value
MOTA	5	ReLink vs. TrackMate	0.011	0.0052 to 0.016	Yes	<0.0001
		ReLink vs. u-Track	0.012	0.0094 to 0.015	Yes	<0.0001
		ReLink vs. MoTT	0.0072	0.0045 to 0.010	Yes	<0.0001
	10	ReLink vs. TrackMate	0.0097	0.0056 to 0.014	Yes	<0.0001
		ReLink vs. u-Track	0.012	0.0093 to 0.014	Yes	<0.0001
		ReLink vs. MoTT	0.013	0.010 to 0.017	Yes	<0.0001
	15	ReLink vs. TrackMate	0.0075	0.0035 to 0.012	Yes	<0.0001
		ReLink vs. u-Track	0.0094	0.0073 to 0.011	Yes	<0.0001
		ReLink vs. MoTT	0.020	0.016 to 0.024	Yes	<0.0001
	20	ReLink vs. TrackMate	0.010	0.0050 to 0.015	Yes	<0.0001
		ReLink vs. u-Track	0.010	0.0071 to 0.013	Yes	<0.0001
		ReLink vs. MoTT	0.027	0.022 to 0.032	Yes	<0.0001
Edge F1	5	ReLink vs. TrackMate	0.0054	0.0027 to 0.0080	Yes	<0.0001
		ReLink vs. u-Track	0.0068	0.0052 to 0.0084	Yes	<0.0001
		ReLink vs. MoTT	0.0029	0.0015 to 0.0043	Yes	<0.0001
	10	ReLink vs. TrackMate	0.0055	0.0033 to 0.0077	Yes	<0.0001
		ReLink vs. u-Track	0.0069	0.0057 to 0.0082	Yes	<0.0001
		ReLink vs. MoTT	0.0063	0.0046 to 0.0081	Yes	<0.0001
	15	ReLink vs. TrackMate	0.0045	0.0021 to 0.0069	Yes	<0.0001
		ReLink vs. u-Track	0.0060	0.0046 to 0.0074	Yes	<0.0001
		ReLink vs. MoTT	0.0091	0.0068 to 0.011	Yes	<0.0001
	20	ReLink vs. TrackMate	0.0057	0.0026 to 0.0089	Yes	0.0002
		ReLink vs. u-Track	0.0065	0.0041 to 0.0088	Yes	<0.0001
		ReLink vs. MoTT	0.013	0.010 to 0.015	Yes	<0.0001

Supplementary Table 1. Dunnett-adjusted comparisons of ReLink with competing linking methods across particle densities. MOTA and Edge F1 scores were evaluated under ideal-localization conditions at particle densities of 5, 10, 15 and 20 particles per frame in a 64 × 64-pixel field of view, corresponding to spatial densities of 1.22, 2.44, 3.66 and 4.88×10^{-3} particles/px^2, respectively. For each metric, data were analyzed using a two-way repeated-measures ANOVA with linking method treated as a within-simulation factor. Sphericity was not assumed, and the Geisser–Greenhouse correction was applied to the omnibus tests. Within each particle density, ReLink was compared with TrackMate, u-Track and MoTT using Dunnett's multiple-comparisons test, with the three comparisons constituting one family (n = 55 simulations per condition). Mean differences were calculated as ReLink minus the indicated comparison method; positive values favor ReLink. Estimated mean differences, simultaneous 95% confidence intervals, and multiplicity-adjusted P values are reported, with adjusted $P < 0.05$ considered statistically significant.

Metrics	SNR	Comparison	Mean Diff.	95% CI	Significant?	Adjusted *P* Value
MOTA	2	ReLink vs. TrackMate	0.023	0.016 to 0.030	Yes	<0.0001
		ReLink vs. u-Track	0.065	0.058 to 0.072	Yes	<0.0001
		ReLink vs. MoTT	0.023	0.020 to 0.027	Yes	<0.0001
	3	ReLink vs. TrackMate	0.036	0.020 to 0.052	Yes	<0.0001
		ReLink vs. u-Track	0.083	0.073 to 0.093	Yes	<0.0001
		ReLink vs. MoTT	0.039	0.036 to 0.043	Yes	<0.0001
	5	ReLink vs. TrackMate	0.037	0.020 to 0.054	Yes	<0.0001
		ReLink vs. u-Track	0.031	0.026 to 0.036	Yes	<0.0001
		ReLink vs. MoTT	0.015	0.0094 to 0.021	Yes	<0.0001
	8	ReLink vs. TrackMate	0.046	0.029 to 0.064	Yes	<0.0001
		ReLink vs. u-Track	0.024	0.018 to 0.029	Yes	<0.0001
		ReLink vs. MoTT	0.014	0.0085 to 0.019	Yes	<0.0001
	15	ReLink vs. TrackMate	0.050	0.031 to 0.068	Yes	<0.0001
		ReLink vs. u-Track	0.029	0.023 to 0.036	Yes	<0.0001
		ReLink vs. MoTT	0.019	0.012 to 0.025	Yes	<0.0001
Edge F1	2	ReLink vs. TrackMate	0.0074	-0.00039 to 0.015	No	0.0664
		ReLink vs. u-Track	0.059	0.053 to 0.064	Yes	<0.0001
		ReLink vs. MoTT	0.0077	0.0049 to 0.011	Yes	<0.0001
	3	ReLink vs. TrackMate	0.017	0.0053 to 0.028	Yes	0.0023
		ReLink vs. u-Track	0.041	0.035 to 0.046	Yes	<0.0001
		ReLink vs. MoTT	-0.013	-0.016 to -0.0088	Yes	<0.0001
	5	ReLink vs. TrackMate	0.022	0.011 to 0.033	Yes	<0.0001
		ReLink vs. u-Track	0.014	0.011 to 0.018	Yes	<0.0001
		ReLink vs. MoTT	0.0044	0.00047 to 0.0083	Yes	0.0244
	8	ReLink vs. TrackMate	0.027	0.016 to 0.039	Yes	<0.0001
		ReLink vs. u-Track	0.011	0.0075 to 0.014	Yes	<0.0001
		ReLink vs. MoTT	0.0038	0.00089 to 0.0066	Yes	0.0072
	15	ReLink vs. TrackMate	0.031	0.019 to 0.043	Yes	<0.0001
		ReLink vs. u-Track	0.015	0.011 to 0.018	Yes	<0.0001
		ReLink vs. MoTT	0.0076	0.0041 to 0.011	Yes	<0.0001

Supplementary Table 2. Dunnett-adjusted comparisons of ReLink with competing linking methods in noisy-video simulations. MOTA and Edge F1 scores were evaluated at signal-to-noise ratios (SNRs) of 2, 3, 5, 8 and 15. Data were analyzed using a two-way repeated-measures ANOVA with linking method as a within-simulation factor. Sphericity was not assumed, and the Geisser–Greenhouse correction was applied to omnibus tests. Within each SNR, ReLink was compared with TrackMate, u-Track and MoTT using Dunnett's multiple-comparisons test, with the three comparisons constituting one family (n = 55 simulations per condition). Mean differences equal ReLink minus the indicated method; positive values favor ReLink, whereas negative values favor the comparison method. Estimated mean differences, simultaneous 95% confidence intervals and multiplicity-adjusted *P* values are reported, with adjusted $P<0.05$ considered statistically significant.

	Comparison	Mean Diff.	95% CI	Significant?	Adjusted *P* Value
w/ Phys	w/ AF vs. w/o AF	0.013	0.0079 to 0.017	Yes	<0.0001
	0.4 vs. w/o AF	0.011	0.0066 to 0.015	Yes	<0.0001
	0.5 vs. w/o AF	0.012	0.0078 to 0.017	Yes	<0.0001
	0.6 vs. w/o AF	0.013	0.0078 to 0.018	Yes	<0.0001
	0.7 vs. w/o AF	0.011	0.0055 to 0.016	Yes	<0.0001
	0.8 vs. w/o AF	0.0051	0.00048 to 0.0097	Yes	0.0246
	0.9 vs. w/o AF	-0.0028	-0.0089 to 0.0033	No	0.7055
w/o Phys	w/ AF vs. w/o AF	0.029	0.020 to 0.038	Yes	<0.0001
	0.4 vs. w/o AF	0.014	0.0092 to 0.019	Yes	<0.0001
	0.5 vs. w/o AF	0.028	0.020 to 0.035	Yes	<0.0001
	0.6 vs. w/o AF	0.034	0.025 to 0.044	Yes	<0.0001
	0.7 vs. w/o AF	0.039	0.028 to 0.051	Yes	<0.0001
	0.8 vs. w/o AF	0.029	0.014 to 0.044	Yes	<0.0001
	0.9 vs. w/o AF	-0.052	-0.075 to -0.029	Yes	<0.0001

Supplementary Table 3. Effects of adaptive and fixed confidence thresholds relative to unfiltered trajectory linking. MOTA was evaluated for ReLink with (w/ Phys) and without (w/o Phys) the physical statistics module under no adaptive filtering (w/o AF), adaptive filtering (w/ AF), and fixed confidence thresholds ranging from 0.4 to 0.9. Data were analyzed using a two-way repeated-measures ANOVA accounting for matched observations across confidence-filtering settings. Sphericity was not assumed, and the Geisser–Greenhouse correction was applied to the omnibus tests. Within each physical-module condition, adaptive filtering and the six fixed thresholds were compared with the corresponding w/o AF condition using Dunnett's multiple-comparisons test, with the seven comparisons constituting one family (n = 55 simulations per condition). Mean differences were calculated as the filtered condition minus w/o AF; positive values indicate improved performance after filtering, whereas negative values indicate reduced performance. Estimated mean differences, simultaneous 95% confidence intervals, and multiplicity-adjusted *P* values are reported, with adjusted $P < 0.05$ considered statistically significant.

Comparison	Threshold	Mean Diff.	95% CI	Significant?	Adjusted *P* Value
w/ Phys vs. w/o Phys	w/o AF	0.040	0.025 to 0.056	Yes	<0.0001
	w/ AF	0.024	0.014 to 0.033	Yes	<0.0001
	0.4	0.037	0.024 to 0.050	Yes	<0.0001
	0.5	0.025	0.013 to 0.036	Yes	<0.0001
	0.6	0.019	0.0098 to 0.028	Yes	<0.0001
	0.7	0.012	0.0035 to 0.021	Yes	0.0014
	0.8	0.016	0.0086 to 0.024	Yes	<0.0001
	0.9	0.089	0.074 to 0.10	Yes	<0.0001

Supplementary Table 4. Effect of the physical statistics module across confidence-filtering settings on MOTA. The same 55 simulations were evaluated with (w/ Phys) and without (w/o Phys) the physical statistics module under no adaptive filtering (w/o AF), adaptive filtering (w/ AF), and fixed confidence thresholds ranging from 0.4 to 0.9. Data were analyzed using a two-way repeated-measures ANOVA with both physical-module status and confidence-filtering setting treated as within-simulation factors. Sphericity was not assumed, and the Geisser–Greenhouse correction was applied to the omnibus tests. Within each filtering setting, w/ Phys was compared with w/o Phys using Bonferroni's multiple-comparisons test, with the eight comparisons constituting one family. Mean differences were calculated as w/ Phys minus w/o Phys; positive values indicate improved performance after inclusion of the physical statistics module. Estimated mean differences, simultaneous 95% confidence intervals, and Bonferroni-adjusted *P* values are reported, with adjusted $P < 0.05$ considered statistically significant.

Metrics	Comparison	SNR	Mean Diff.	95% CI	Significant?	Adjusted *P* Value
MOTA	w/ AF vs. w/o AF	5	0.0069	0.0056 to 0.0082	Yes	<0.0001
		15	0.0058	0.0043 to 0.0072	Yes	<0.0001
Edge F1	w/ AF vs. w/o AF	5	0.0037	0.0025 to 0.0050	Yes	<0.0001
		15	0.0044	0.0030 to 0.0059	Yes	<0.0001

Supplementary Table 5. Effect of adaptive confidence filtering in challenging video-based simulations. MOTA and Edge F1 scores were evaluated for ReLink with adaptive filtering (w/ AF) and without adaptive filtering (w/o AF) for challenging video-based simulations at SNR = 5 and SNR = 15 from Fig. 2. For each metric, data were analyzed using a two-way ANOVA with filtering status treated as a matched, two-level within-simulation factor. No sphericity correction was required because this repeated factor contained only two levels. Within each SNR, w/ AF was compared with w/o AF using Bonferroni's multiple-comparisons test, with the two SNR-specific comparisons constituting one family (n = 30 simulations at SNR = 5 and n = 24 simulations at SNR = 15). Mean differences were calculated as w/ AF minus w/o AF; positive values indicate improved trajectory-linking performance after adaptive filtering. Estimated mean differences, simultaneous 95% confidence intervals, and Bonferroni-adjusted *P* values are reported, with adjusted $P < 0.05$ considered statistically significant.

Metrics	Comparison	Diffusion Coefficient (px^2/frame)	Mean Diff.	95% CI	Significant?	Adjusted *P* Value
MOTA	w/ AF vs. w/o AF	10^{1}	0.017	0.012 to 0.023	Yes	<0.0001
		$10^{1.25}$	0.020	0.015 to 0.026	Yes	<0.0001
		$10^{1.5}$	0.034	0.028 to 0.040	Yes	<0.0001
Edge F1	w/ AF vs. w/o AF	10^{1}	0.010	0.0042 to 0.016	Yes	0.0001
		$10^{1.25}$	0.014	0.0086 to 0.020	Yes	<0.0001
		$10^{1.5}$	0.027	0.022 to 0.033	Yes	<0.0001

Supplementary Table 6. Effect of adaptive confidence filtering under fast-diffusion conditions. MOTA and Edge F1 scores were evaluated for ReLink with adaptive filtering (w/ AF) and without adaptive filtering (w/o AF) at diffusion coefficients of 10^{1}, $10^{1.25}$ and $10^{1.5}$ px^2/frame. For each metric, data were analyzed using a two-way ANOVA with filtering status treated as a matched, two-level within-simulation factor. No sphericity correction was required because this repeated factor contained only two levels. Within each diffusion coefficient, w/ AF was compared with w/o AF using Bonferroni's multiple-comparisons test, with the three diffusion-specific comparisons constituting one family (n = 55 simulations per condition). Mean differences were calculated as w/ AF minus w/o AF; positive values indicate improved performance after adaptive filtering. Estimated mean differences, simultaneous 95% confidence intervals, and Bonferroni-adjusted *P* values are reported, with adjusted $P < 0.05$ considered statistically significant.

	W1 (α)	W1 (D)	RMSE (CP)	JSC (CP)	MAE (α)	MSLE (D)
TrackMate	0.249	0.066	3.396	0.326	0.397	0.390
ReLink (w/o AF)	0.214	0.060	2.779	0.448	0.236	0.181
ReLink (w/ AF)	0.211	0.059	2.705	0.443	0.243	0.167
ReLink (τ=0.4)	0.213	0.061	2.716	0.447	0.242	0.165
ReLink (τ=0.5)	0.211	0.060	2.710	0.447	0.241	0.162
ReLink (τ=0.6)	0.212	0.058	2.699	0.440	0.243	0.166
ReLink (τ=0.7)	0.214	0.057	2.749	0.439	0.244	0.182
ReLink (τ=0.8)	0.210	0.055	2.784	0.430	0.252	0.169
ReLink (τ=0.9)	0.207	0.039	2.999	0.415	0.262	0.173

Supplementary Table 7. Comparison of evaluation metrics under different confidence thresholds for AnDi 2 Benchmark. For the Ensemble Task, Wasserstein-1 distances for the anomalous exponent (W1 (α)) and diffusion coefficient (W1 (D)) are reported. For the Single-Trajectory Task, metrics include RMSE for changepoint detection (RMSE (CP)), Jaccard similarity coefficient for changepoint detection (JSC (CP)), mean absolute error for anomalous exponent (MAE (α)) and mean squared logarithmic error for diffusion coefficient (MSLE (D)). Results are shown for trajectories reconstructed by TrackMate, ReLink without confidence filtering, ReLink with fixed confidence thresholds τ ranging from 0.4 to 0.9, and ReLink with the adaptive filtering.

Dataset	Max linking distance	Max gap length
AnDi 1 Benchmark	15	2
AnDi 2 Benchmark	20	5
Cell Tracking Challenge (no gap-closing)	/	/
Glycerol-water mixtures	20	5
Hydrogel gelation	20	5
L929 Cell lines	15	5

Supplementary Table 8. Post-processing parameters for trajectory reconstruction.

Module	Parameter	Value
Input	Raw node feature dimension	3
	Raw edge feature dimension	5
Graph Attention Network	Hidden feature dimension	128
	Number of attention heads	4
Transformer	Number of encoder layers	4
	Number of heads	4
	Hidden feature dimension	128
Score Net	Input feature dimension	3
	Hidden feature dimension	16
	Output feature dimension	128
Histogram Net	Histogram dimension of each FiLM network	10
	FiLM parameter dimension (γ and β)	256
Dropout	Rate	0.3
Optimizer	AdamW learning rate	1e-3

Supplementary Table 9. Network architecture and training hyperparameters fixed across all datasets. The raw node feature dimension is 3, corresponding to the two-dimensional spatial coordinates and frame index (x, y, t). The raw edge feature dimension is 5, corresponding to the per-axis displacements, temporal separation, Euclidean distance and speed $(\Delta x, \Delta y, \Delta t, d, v)$. The Score Net receives the three lag-specific local-to-global CDF scores computed at $\Delta t = 1,2,3$ and maps them to a 128-dimensional embedding. Each local or global FiLM network receives a 10-bin jump-distance histogram and outputs a 256-dimensional vector containing the concatenated modulation parameters $\gamma \in \mathbb{R}^{128}$ and $\beta \in \mathbb{R}^{128}$. The same architectural dimensions and training hyperparameters were used for all datasets.

**Supplementary Algorithm 1**	Adaptive confidence filtering with Monte Carlo dropout and KDE valley threshold
**Input:**	all candidate edges $\mathcal{E}$, trajectory linking model $f(\cdot;\theta)$, number of Monte Carlo dropout[8] samples N, KDE grid size G, numerical-stability constant ϵ
**Output:**	confidence $\{c_e\}$, adaptive threshold τ^*, kept edges E_{keep}
**1.**	for each edge $e \in \mathcal{E}$:
**2.**	for $n = 1 \dots N$:
**3.**	sample $w_n \sim q_\theta(w)$ using Monte Carlo dropout
**4.**	compute $z_e^{(n)} = f\left(e;\, w^{(n)}\right); p_e^{(n)} = \sigma\left(z_e^{(n)}\right)$
**5.**	compute $\hat{p}_e = \sum p_e^{(n)} / N$; clamp $\hat{p}_e\ to\ [\epsilon, 1-\epsilon]$
**6.**	compute $H(\hat{p}_e) = -\hat{p}_e \log_2 \hat{p}_e - (1-\hat{p}_e)\log_2(1-\hat{p}_e)$
**7.**	set confidence $c_e = 1 - H(\hat{p}_e)$
**8.**	Collect $\{c_e\}_{e\in\mathcal{E}}$
**9.**	Compute the sample standard deviation sd_c of $\{c_e\}$ and set $\sigma_{\mathrm{bw}} = \mathrm{sd}_c|\mathcal{E}|^{-1/5}$
**10.**	Fit KDE $\hat{f}(c)$ to the confidence scores $\{c_e\}$
**11.**	Evaluate $\hat{f}$ on a grid of G points; detect two dominant peaks (c_L, c_R)
**12.**	Find adaptive threshold: $\tau^* = argmin_{c\in(c_L,c_R)} \hat{f}(c)$
**13.**	Filter: $\mathrm{E}_{keep} = \{e \mid c_e \geq \tau^*\}$
**14.**	Return $\{c_e\}, \tau^*, \mathrm{E}_{keep}$

Supplementary Note 1. Split event detection strategy in post-processing stage

After trajectory reconstruction, we further perform a post-processing step to detect cell-division (split) events, which are common in the Cell Tracking Challenge dataset but are not explicitly modeled during the initial linking stage. Given the reconstructed trajectories, we examine whether a single trajectory may branch into two daughter trajectories at an intermediate time point. Specifically, for each reconstructed trajectory $\mathrm{Traj}_A = (v_1, v_2, \ldots, v_L)$, we consider its intermediate nodes $u = v_i (1 < i < L)$, which already have an outgoing connection to the next node v_{i+1}. We then search for alternative candidate connections from u to the start node v of another trajectory T_B. If such a candidate exists, we retrieve the predicted probabilities for the two outgoing links: $p_1 = p(u \rightarrow v_{i+1})$, corresponding to the original continuation of Traj_A, and $p_2 = p(u \rightarrow v)$, corresponding to the potential initiation of Traj_B. Rather than applying a hard threshold on the alternative link probability (e.g. $p_2 > 0.5$), we quantify the relative deviation between the two competing outgoing links using the difference $(p_1 - p_2)/p_1$. A split event is detected when this quantity is smaller than a predefined threshold, indicating that the network assigns comparable confidence to both outgoing connections from the same parent node. Intuitively, this condition captures the scenario in which the alternative link is not significantly worse than the existing link, suggesting a likely division event rather than a spurious association. To avoid over-fragmentation, we allow at most one split per trajectory and retain the split candidate that yields the minimal relative deviation. Once a split is confirmed, the parent trajectory is terminated at node u, and two daughter trajectories are formed by following the two outgoing links. This relative-deviation-based criterion provides a scale-invariant and more interpretable signal for split detection, making it particularly suitable for dense and noisy cell tracking scenarios.

Supplementary Note 2. Detailed parameter settings for generating simulated datasets

For training, we generate two-dimensional FBM multi-state diffusion trajectories using *andi-datasets* package[9], where each trajectory samples an anomalous exponent $\alpha \sim U(0, 2)$ and a diffusion coefficient $D \sim (10^{-1}, 10^{1})$. Each sample contains 50 frames within a field of view of 64×64 pixels, and an average of 20 particles per FOV. A total of 55 samples are generated for training.

For localization-based test datasets, we use the same sampling distributions for α and D as in training data generation, but vary several experimental factors: (1) Max trajectory lengths: 20, 50, 100, 150, 200; (2) Particle counts per FOV: 5, 10, 15, 20; (3) FOV sizes: 64, 128, 256, 512, 1024 pixels; (4) Diffusion models: single-state, multi-state, confinement, dimerization, immobilization. These combinations enable systematic evaluation across different motion types, densities, timescales and spatial resolutions.

For video-based simulations, we generate two-dimensional multi-state diffusion trajectories using the same sampling distributions for the anomalous exponent (α) and diffusion coefficient (D) as in the training data. Simulated videos contain 50 frames, with the average particle count set to 5, 10, 15 or 20 particles/frame under different simulation conditions. Each video is rendered from particle trajectories using the DeepTrack fluorescence optical model, with particles represented as point emitters. The optical parameters include a wavelength of 500 nm, a numerical aperture of 1.46 and an effective pixel size of 100 nm/pixel. For an ideal diffraction-limited point spread function (PSF), these parameters correspond to a theoretical lateral full width at half maximum (FWHM) of approximately 176 nm (1.76 pixels) before pixel sampling. Emitter intensities are independently sampled from a Gaussian distribution with a standard deviation of 20 in simulator intensity units. The mean emitter intensity is adjusted to obtain nominal signal-to-noise ratio (SNR) levels of 2, 3, 5, 8 and 15, while the uniform background level remains fixed at 100. Each emitter retains its sampled intensity throughout its trajectory, without additional blinking or photobleaching. Poisson noise is applied, and the simulated frames are individually normalized and converted to 8-bit images.

For data used in Fig. 3a–d, to highlight the effectiveness of confidence estimation under fast-diffusion and challenging scenarios, we purposely increase task difficulty by generating multi-state trajectories with anomalous exponent $\alpha \sim U(0, 2)$ and high diffusion coefficients, sampled from $D \sim U(10^{0}, 10^{1})$, while frames and density remain 50 and 15 respectively. This setup enlarges the performance gap between models with and without the adaptive filtering module, facilitating clearer comparison. For Fig. 3f, we extend the above dataset by adding confinement and dimerization models to evaluate confidence prediction effectiveness across motion types. To further test performance under ultra-fast diffusion, Fig. 3g includes additional datasets with $10^{1}, 10^{1.25}, 10^{1.5}$ px^2/frame, representing increasingly challenging linking conditions.

Supplementary Note 3. Detailed settings for public benchmark datasets

For the AnDi 2 Benchmark evaluation, we directly use the official dataset from the AnDi 2 Challenge[10]. This benchmark consists of nine experiments spanning: single-state, multi-state, dimerization, quenched-trap, transient-confinement model. Each experiment provides ground-truth diffusion coefficients, anomalous exponents, and model-specific parameters. The whole task includes: (1) particle localization from video frames, (2) trajectory linking, and (3) analysis of each trajectory (changepoint detection, segment-wise D and α estimation) and ensemble-level inference of D and α distributions from 30 FOVs per experiment. We follow the official AnDi 2 evaluation metrics to ensure consistency with leaderboard results.

For AnDi 1 dataset, following the task definitions of the AnDi 1 Challenge[9], we construct a dataset with five diffusion models: annealed transient time motion (ATTM), continuous-time random walk (CTRW), fractional Brownian motion (FBM), Lévy walk (LW) and scaled Brownian motion (SBM). Each model contains five videos, with 200 frames and 32 particles per video, each exhibiting a distinct anomalous exponent α. Performance is assessed using F1 score for diffusion-model classification and MAE for anomalous-exponent regression.

For the Cell Tracking Challenge datasets[11], we follow the procedure adopted in MAGIK[12]. Cell localization files (.csv) and division-event annotations (parenthood.csv) for HeLa and MuSC cells are obtained from the DeepTrack package[13]. Division relationships are extracted by matching the trajectory ID of each parent cell with the trajectory IDs of its two daughter cells as specified in the parenthood.csv file. Using this information, we explicitly link the cell localization of parent trajectory at the division time point to each of the two daughter trajectories, thereby constructing two new child trajectories originating from the division event. In this formulation, each division event is represented by three trajectories, including one parent trajectory up to the division point and two child trajectories starting from division point, which together define the ground-truth trajectories. This representation enables straightforward evaluation of trajectory-linking performance using standard multi-object tracking metrics, including MOTA.

Supplementary Note 4. Home-built microscope for single-particle tracking

The configuration of the home-built microscope is shown in Supplementary Fig. 15. The system is based on an inverted wide-field fluorescence microscope and supports multichannel excitation as well as multiple illumination modes.

(1) Optical setup

The microscope is equipped with three single-mode laser channels at wavelengths of 405 nm, 561 nm, and 637 nm. After collimation, spectral filtering, and beam expansion, the laser outputs are used as excitation light sources. The excitation beam is subsequently focused onto the back focal plane of the objective. A translation stage is implemented to precisely control the position of the focal spot within the back focal plane, enabling different illumination modalities, including epifluorescence (EPI), total internal reflection (TIRF), and highly inclined laminated optical sheet (HILO) illumination. The sample with the diffusing tracer particles is mounted on an XYZ piezoelectric stage (PIEZOCONCEPT, LFHS3.50.50) for fine positioning and system calibration. Fluorescence emission from the sample is separated from the excitation light using a dichroic mirror and is finally detected by an EMCCD camera for signal acquisition.

(2) Experimental configuration and imaging conditions

Prior to experiments, the imaging system was calibrated using the XYZ piezoelectric stage to determine the effective pixel size, which was around 130 nm per pixel. All experiments were conducted with a fixed field of view of 512 × 512 pixels at a frame rate of 20 fps. For measurements of fluorescent bead motion in glycerol–water mixtures and hydrogels, excitation was performed using the 637 nm laser, and HILO illumination was employed to minimize out-of-focus background fluorescence. For observations of quantum dot diffusion in L929 cells, the 637 nm laser was first used to excite membrane-staining fluorophores to acquire cell images. Subsequently, the 405 nm and 561 nm lasers were used to excite quantum dots for SPT video acquisition. Both cell images and SPT videos were acquired under epifluorescence illumination. During data analysis, only intracellular regions were selected for particle localization, trajectory linking, and subsequent quantitative analysis.

Supplementary Note 5. Experimental protocols

This note details sample preparation, cell culture and labeling/delivery for the hydrogel-gelation and L929 datasets referenced in Methods (Experimental data).

Thermal curing test of the hydrogel: 1) Prepare a hydrogel monomer solution made of 4% N.N-dimethylacrylamide (v/v), 34% Sodium acrylate (w/v), 10% Acrylamide (w/v), 0.005% N.N'-Methylenebisacrylamide (w/v), 1% Sodium chloride (w/v), and 1× PBS and stored at 4 °C before synthesis. 2) During the curing test, 200 nm fluorescent microspheres (Invitrogen™,Carboxylate-Modified Microspheres) are added at a 1:4000 dilution and mixed thoroughly, with all subsequent steps performed under light-protected conditions. To enable rapid hydrogel curing, the monomer solution is generally supplemented with 1% (w/v) ammonium persulfate and 1% (v/v) tetramethylethylenediamine, followed by prompt mixing. The hydrogel monomer solution containing the thermal initiators is then quickly dropped onto a glass slide, gently covered with a coverslip, and immediately transferred to the microscope for imaging.

Cell culture: Cell lines L929 were grown in high glucose Dulbecco's Modified Eagle Medium (DMEM, Gibco,10566) supplemented with GlutaMAX, supplemented with 10% Fetal Bovine Serum (FBS, Gibco A5256701) and penicillin and streptomycin (100 µg/ml) at 37°C in a humidified 5% CO2 incubator. Cells were used with passage numbers below 20 and free of mycoplasma contamination.

Electroporation-delivered quantum dots: 1) Preparation of Cells: One day prior to electroporation, detach adherent cells via trypsin digestion and transfer to a culture dish containing fresh growth medium. The seeding density should ensure that the cells reach 50%–70% confluence on the day of electroporation. Before electroporation, remove the growth medium and wash the cells with PBS, then digestion cell again using trypsin. Collect the trypsinized cell suspension and determine the cell density using a hemocytometer. Centrifuge the cell suspension at 1000×g for 3 minutes at room temperature. 2) Electroporation: Resuspend the cell pellet in an appropriate pre-cooled sterile electroporation buffer (8 mM Na2HPO4, 2 mM KH2PO4, 1 mM MgSO4 and 250 mM sucrose，pH 7.4) at a density of 1×10^6 to 5×10^6 cells/ml. Gently pipette the mixture to obtain a homogeneous suspension. Set the parameters on the electroporation instrument (Bio-Rad MicroPulser Electroporator, 1652100) to 220 V and a pulse duration of 4 ms. Allow the cell suspension to rest on ice for 5 minutes to reduce endocytosis, then dilute and add quantum dot solution (Qdot™ 655 ITK™ carboxyl quantum dot) at a 1:160,000 or 1:320,000 ratio. After mixing, transfer the suspension into an electroporation cuvette. Place the cuvette into the electroporator and deliver two pulses. Then, let the entire cuvette stand at room temperature for 7 minutes to promote cell membrane recovery. Subsequently, add fresh medium into the electroporation cuvette. Centrifuge at 1000×g for 3 minutes at room temperature. Remove the supernatant and resuspend the cells in PBS. Repeat centrifugation at 1000×g for 3 minutes at room temperature. Remove the PBS, resuspend the cells in fresh medium, and transfer them to a confocal dish. Culture the cells in a humidified incubator at 37°C with 5% CO_2 for 12 hours.

Reference

1. Y. Zhang, and G. Yang, “A Motion Transformer for Single Particle Tracking in Fluorescence Microscopy Images,” in *Medical Image Computing and Computer Assisted Intervention – MICCAI 2023*, edited by H. Greenspan, A. Madabhushi, P. Mousavi, et al., Vol. 14227, Springer Nature Switzerland: Cham 2023, pp. 503–513, https://doi.org/10.1007/978-3-031-43993-3_49.

2. J.-Y. Tinevez, N. Perry, J. Schindelin, et al., “TrackMate: An Open and Extensible Platform for Single-Particle Tracking,” *Methods 115* (2017): 80–90, https://doi.org/10.1016/j.ymeth.2016.09.016.

3. K. Jaqaman, D. Loerke, M. Mettlen, et al., “Robust Single-Particle Tracking in Live-Cell Time-Lapse Sequences,” *Nature Methods 5*, no. 8 (2008): 695–702, https://doi.org/10.1038/nmeth.1237.

4. X. Feng, Y. Jiang, H. Sha, et al., “Enhancing Diffusion Estimation in Single-Particle Experiments through Motion Change Analysis Using Deep Learning,” *Journal of Physics: Photonics 7*, no. 3 (2025): 035006, https://doi.org/10.1088/2515-7647/add978.

5. P. Veličković, G. Cucurull, A. Casanova, A. Romero, P. Liò, and Y. Bengio, “Graph Attention Networks,” in *International Conference on Learning Representations*, 2018.

6. A. Vaswani, N. Shazeer, N. Parmar, et al., “Attention Is All You Need,” in *Advances in Neural Information Processing Systems*, Vol. 30, 2017, pp. 5998–6008.

7. E. Perez, F. Strub, H. De Vries, V. Dumoulin, and A. Courville, “FiLM: Visual Reasoning with a General Conditioning Layer,” in *Proceedings of the AAAI Conference on Artificial Intelligence*, Vol. 32, 2018, pp. 3942–3951, https://doi.org/10.1609/AAAI.V32I1.11671.

8. Y. Gal, and Z. Ghahramani, “Dropout as a Bayesian Approximation: Representing Model Uncertainty in Deep Learning,” in *Proceedings of the 33rd International Conference on Machine Learning*, Vol. 48, PMLR 2016, pp. 1050–1059.

9. G. Muñoz-Gil, G. Volpe, M. A. Garcia-March, et al., “Objective Comparison of Methods to Decode Anomalous Diffusion,” *Nature Communications 12*, no. 1 (2021): 6253, https://doi.org/10.1038/s41467-021-26320-w.

10. G. Muñoz-Gil, H. Bachimanchi, J. Pineda, et al., “Quantitative Evaluation of Methods to Analyze Motion Changes in Single-Particle Experiments,” *Nature Communications 16*, no. 1 (2025): 6749, https://doi.org/10.1038/s41467-025-61949-x.

11. V. Ulman, M. Maška, K. E. Magnusson, et al., “An Objective Comparison of Cell-Tracking Algorithms,” *Nature Methods 14*, no. 12 (2017): 1141–1152, https://doi.org/10.1038/nmeth.4473.

12. J. Pineda, B. Midtvedt, H. Bachimanchi, et al., “Geometric Deep Learning Reveals the Spatiotemporal Features of Microscopic Motion,” *Nature Machine Intelligence 5*, no. 1 (2023): 71–82, https://doi.org/10.1038/s42256-022-00595-0.

13. B. Midtvedt, S. Helgadottir, A. Argun, J. Pineda, D. Midtvedt, and G. Volpe, “Quantitative Digital Microscopy with Deep Learning,” *Applied Physics Reviews 8*, no. 1 (2021): 011310, https://doi.org/10.1063/5.0034891.
